# Cell cycle–dependent centrosome clustering precedes proplatelet formation

**DOI:** 10.1126/sciadv.adl6153

**Published:** 2024-06-19

**Authors:** Isabelle C. Becker, Adrian R. Wilkie, Emma Nikols, Estelle Carminita, Harvey G. Roweth, Julia Tilburg, Anthony R. Sciaudone, Leila J. Noetzli, Farheen Fatima, Genevieve Couldwell, Anjana Ray, Alex Mogilner, Kellie R. Machlus, Joseph E. Italiano

**Affiliations:** ^1^ Vascular Biology Program, Boston Children’s Hospital, 1 Blackfan Circle, Boston, MA 02115, USA.; ^2^ Harvard Medical School, 25 Shattuck Street, Boston, MA 02115, USA.; ^3^ Brigham and Women’s Hospital, 4 Blackfan Circle, Boston, MA 02115, USA.; ^4^ Courant Institute of Mathematical Sciences, New York University, 251 Mercer Street, New York, NY 10012, USA.

## Abstract

Platelet-producing megakaryocytes (MKs) primarily reside in the bone marrow, where they duplicate their DNA content with each cell cycle resulting in polyploid cells with an intricate demarcation membrane system. While key elements of the cytoskeletal reorganizations during proplatelet formation have been identified, what initiates the release of platelets into vessel sinusoids remains largely elusive. Using a cell cycle indicator, we observed a unique phenomenon, during which amplified centrosomes in MKs underwent clustering following mitosis, closely followed by proplatelet formation, which exclusively occurred in G_1_ of interphase. Forced cell cycle arrest in G_1_ increased proplatelet formation not only in vitro but also in vivo following short-term starvation of mice. We identified that inhibition of the centrosomal protein kinesin family member C1 (KIFC1) impaired clustering and subsequent proplatelet formation, while KIFC1-deficient mice exhibited reduced platelet counts. In summary, we identified KIFC1- and cell cycle–mediated centrosome clustering as an important initiator of proplatelet formation from MKs.

## Teaser

Megakaryocytes cluster their centrosomes before proplatelet formation, a phenomenon that can be enhanced upon cell cycle arrest.

## INTRODUCTION

Platelets are derived from large progenitors, megakaryocytes (MKs), that predominantly reside within the bone marrow (BM) (*1*). During their development from hematopoietic stem cells, MKs increase mRNA and protein synthesis, accumulate specialized granules, and develop an invaginated membrane reservoir [demarcation membrane system (DMS)] required for platelet production (*2*). MKs then extrude long protrusions called proplatelets, each composed of platelet-sized swellings, into vessel sinusoids (*3*, *4*), where they undergo additional morphological changes to give rise to circulating platelets (*2*, *5*). MKs express a lineage-specific isoform of β1-tubulin, which is posttranslationally modified to mediate proplatelet formation (*6*–*9*). Before proplatelet production, microtubules are distributed throughout the MK cytoplasm. However, once proplatelets form, microtubules line the cell cortex, where dynein-dependent microtubule-sliding forces promote elongation (*10*). How this redistribution of microtubules occurs has not been thoroughly investigated to date. Moreover, while important molecular mediators of proplatelet elaboration and release have been identified in recent years (*11*), what directly induces MKs to release proplatelets remains unknown.

In addition to proplatelet formation, recent studies have suggested alternative mechanisms of platelet production via direct budding of platelets from the MK surface (*12*) or the release of larger membrane protrusions that further mature within the vasculature (*13*). Moreover, during emergency thrombopoiesis, e.g., upon induction of thrombocytopenia, MKs can rupture to induce a more rapid release of platelet-like particles (*14*). However, in line with proplatelet formation, mechanistic knowledge of the signaling trajectories initiating these potential alternative forms of platelet release remains elusive.

Thrombocytopenia (platelet counts below 150 × 10^3^ μl^−1^) is encountered across a variety of disorders including immune thrombocytopenic purpura (ITP), myelodysplastic syndrome, and various genetic conditions as well as induced by drugs during chemotherapy (*15*, *16*). Thrombopoietin (TPO) mimetics are administered to patients, promoting the differentiation of hematopoietic precursors into MKs; however, they take 5 to 12 days to increase platelet counts, and a variety of patients have reduced counts despite normal MK numbers (*17*–*19*). Therefore, therapeutics that directly and acutely enhance platelet production from existing MKs are urgently needed.

In contrast to almost all other cell types, MKs can bypass the final stages of the cell cycle in a process termed endomitosis; chromosomes condense and align in metaphase, but MKs do not progress to cytokinesis, effectively doubling their DNA content with each endomitotic cycle (*20*). The cell cycle in mammalian cells is typically divided into two phases: interphase, consisting of G_1_, S, and G_2_, and mitosis (M), in which duplicated chromosomes are separated (*21*). Past studies have identified both cell cycle and cytoskeletal proteins as endomitotic regulators (*22*–*25*). Trakala and colleagues (*26*) highlighted that mitotic arrest in vivo prevented megakaryopoiesis and thrombopoiesis resulting in severe thrombocytopenia. Inhibition of mitotic regulators on the other hand enabled endoreduplication in MKs—a mechanism circumventing endomitosis—which still allowed for normal MK development and platelet production, suggesting a possible uncoupling of MK maturation and endomitosis. How and whether the cell cycle directly influences (pro)platelet production, however, has not been investigated. Notably, endomitosis also leads to the amplification of centrosomes, which can serve as microtubule organizing centers (MTOCs) during interphase and mitosis and can promote microtubule nucleation (*27*).

Centrosomes consist of two centrioles in perpendicular orientation and the pericentriolar material (*28*). Amplified centrosomes in diploid or tetraploid cancer cells can cluster, thus preventing aneuploidy by consolidation into a bipolar spindle (*29*). Centrosome clustering is dependent on the activity of a plethora of centrosomal proteins such as aurora kinases, c-terminally encoded peptide (CEP) family proteins, and the Augmin complex (*30*), as well as microtubule regulators such as kinesin family members and dynein (*31*, *32*). Recently, centrosome clustering has also been identified in noncancerous, polyploid cells such as osteoclasts and dendritic cells, where it induces cytoskeletal remodeling (*33*, *34*). Because of its unique occurrence in polyploid or aneuploid cells, there have been substantial efforts toward developing therapies that inhibit centrosome clustering and ultimately cancer cell viability. A variety of genetic and small-molecule inhibitor studies have converged on kinesin family member C1 (KIFC1; also known as HSET) as a promising target due to its essential role in centrosome clustering in cancer cells (*35*–*37*). KIFC1 is a member of the kinesin-14 family and primarily localizes to the mitotic spindle, where its minus-end-directed movement on microtubules helps maintain spindle symmetry during metaphase (*38*). KIFC1 levels are frequently elevated in cancer cells, which is thought to promote centrosome clustering and bipolar spindle assembly by merging microtubules from separate centrosomes (*35*, *39*). In contrast, inhibition of KIFC1 did not only cause mitotic catastrophe (*40*) but also hindered DNA synthesis during S phase, leading to increased cell death due to the formation of micronuclei in daughter cells after mitosis (*41*).

In this study, we found that MKs cluster their centrosomes following mitosis, a phenomenon that persisted throughout subsequent cell cycle stages, while proplatelets were exclusively formed in G_1_. G_1_ cell cycle arrest manifested supercentrosome and proplatelet formation in vitro and mice undergoing short-term starvation (STS) for up to 48 hours displayed a significant increase in platelet production. Ultimately, we identified the kinesin family protein KIFC1 as a central regulator of centrosome clustering in MKs in vitro and in vivo. In summary, we describe the cell biological phenomenon of centrosome clustering in human and murine MKs, a pathway that may be targeted therapeutically to modulate platelet counts.

## RESULTS

### The cell cycle governs centrosomal clustering and proplatelet formation in MKs

**Fig. 1.**
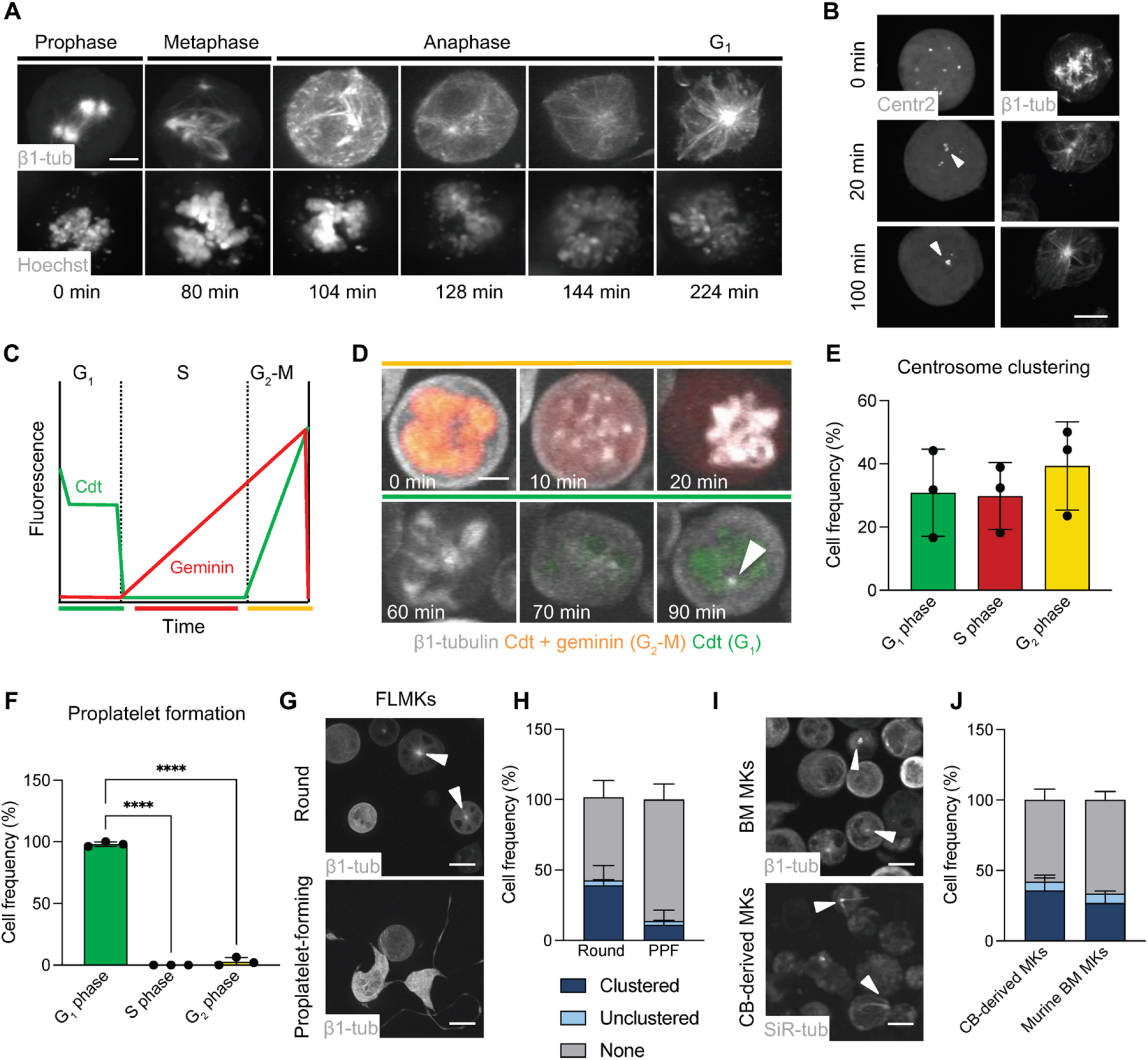
Cell cycle dynamics regulate centrosome clustering and proplatelet formation in murine MKs. (**A**) Visualization of β1-tubulin and DNA (Hoechst) during the different stages of mitosis in FLMKs transduced with MSCV–β1-tubulin–dendra2 before proplatelet formation. Live-cell image acquisition was performed every 10 min. One representative cell is shown. Scale bar, 10 μm. (**B**) Visualization of centrosome clustering in MSCV-centrin2-GFP– and MSCV–β1-tubulin–dendra2–transduced FLMKs following mitosis. Images were acquired as described above. Scale bar, 10 μm. White arrowheads point at supercentrosomes. (**C**) Differential gene expression of MSCV-FUCCI cell cycle reporter: mCherry (geminin, red) and/or mVenus (cdt, green) are expressed depending on the cell cycle stage. (**D**) Visualization of FUCCI expression cycles in FLMKs stained with SiR-tubulin. Image acquisition was performed every 10 min. Scale bar, 8 μm. Arrowhead highlights clustered centrosome. (**E** and **F**) Percentage of cells in different cell cycle stages during supercentrosome formation (E) and proplatelet formation (F). *n* = 3. At least 200 cells were analyzed per mouse. Data are presented as means ± SD. One-way analysis of variance (ANOVA) with Sidak correction for multiple comparisons. ****P < 0.0001. Colors coordinate with FUCCI expression. (**G** and **H**) Visualization and quantification of centrosome clustering in murine FLMKs transduced with MSCV–β1-tubulin–dendra2 before and during proplatelet formation (PPF). *n* = 3. At least 200 cells were analyzed per mouse. Data are presented as means ± SD. Arrowheads highlight clustered centrosome. (**I** and **J**) Visualization and quantification of centrosome clustering in murine BM MKs (top) and human umbilical cord blood (CB)–derived MKs. *n* = 3. At least 200 cells were analyzed per sample. Data are presented as means ± SD.

Although highly controlled cell cycle regulation is vital during MK differentiation (*42*), whether proplatelet formation is a cell cycle–dependent process has not been investigated. To examine this, we optimized a method to visualize fluorescently tagged β1-tubulin in live MKs (*43*). murine stem cell virus (MSCV)–β1-tubulin–dendra2–transduced fetal liver–derived MKs (FLMKs) were stained with Hoechst and imaged over several hours to investigate cell cycle dynamics before and during proplatelet formation (Fig. 1A). Notably, similar to previous experiments (*43*), transduction efficiencies for all used MSCV vectors ranged between 20 and 40% (fig. S1, A and B). Using this approach, we observed an unexpected clustering of amplified centrosomes following the alignment of multipolar spindles with condensed DNA in metaphase, from which microtubules emanated and entered protrusions reminiscent of proplatelets (Fig. 1A, rightmost, and fig. S1C). To directly visualize centrosomes, we next transduced FLMKs with MSCV–centrin2–green fluorescent protein (GFP), which is similar to pericentrin (*44*), one of several centriolar proteins localizing to centrosomes (*45*). We observed that individual centrosomes initially appeared as separate components of a multipolar spindle during mitosis and then clustered into one large structure upon entry into G_1_ (Fig. 1B and movie S1). We termed these so-far undescribed clusters of numerous centrosomes localizing to the cytoplasmic center of the cell “supercentrosomes.”

To identify whether the cell cycle stage was relevant for centrosome clustering, we induced the expression of a fluorescence ubiquitination cell cycle indicator (MSCV-PIP-FUCCI) in FLMKs (*46*). Transduced MKs expressed proteins fused to mCherry (geminin, red) and/or mVenus (Cdt, green) depending on their cell cycle stage (Fig. 1C). Centrosomes began to cluster following mitosis; however, supercentrosome formation only fully commenced once MKs entered into G_1_ of interphase (Fig. 1D, white arrowhead, and movie S2). Classification of MKs for each cell cycle stage revealed that supercentrosomes were apparent in nearly equal proportions for each cell cycle stage following G_1_ (Fig. 1E), collectively suggesting that supercentrosomes are formed during G_1_ but can persist throughout subsequent cell cycle stages until mitosis, in which centrosomes decluster to form a multipolar spindle. Continuous imaging of MKs until proplatelet formation revealed that, virtually, all proplatelet-forming MKs were green, i.e., in G_1_ of interphase (Fig. 1F and movie S3), suggesting that proplatelet formation is a cell cycle–dependent process.

Overall, supercentrosomes were more prevalent in MKs before proplatelet formation (39 ± 14%; movie S4) than in MKs actively making proplatelets (11 ± 10%; Fig. 1, G and H, and movie S5). Most frequently, we observed clustered centrosomes immediately before proplatelet formation (fig. S1C and movies S6 and S7). When correlating centrosome clustering to ploidy, we found no difference in nuclear area between MKs with unclustered versus clustered centrosomes; however, we observed a higher nuclear ratio in comparison to cell size in MKs with active clustering, suggesting that a specific subset of MKs utilizes clustering (fig. S1, D and E). Notably, similar structures were apparent in murine BM MKs and human umbilical cord blood–derived MKs stained with silicon rhodamine (SiR)-tubulin (Fig. 1, I and J), suggesting that supercentrosome formation in MKs was conserved between species.

A balance of protein translation and degradation within MKs is vital during platelet production (*47*). Along these lines, polysome profiling of MKs before and during proplatelet formation has previously revealed a specific subset of proteins that are translated upon initiation of platelet production, several of which are involved in cell cycle or centrosome regulation (table S1) (*48*). Since cell cycle and centrosomal dynamics are highly dependent on a distinct up- and down-regulation of proteins, we next tested whether we could identify a similar protein signature when analyzing proteins targeted for degradation upon proplatelet formation. To this end, we performed a ubiquitin pull-down assay on MKs treated with the proteasome inhibitor MG132, which was previously shown to inhibit proplatelet formation (*47*). MKs were enriched and treated with vehicle control or MG132 (to prevent protein degradation) for 4 hours, after which ubiquitinated proteins were isolated and analyzed. In addition to the previously identified accumulation of the cytoskeletal guanosine triphosphatase RhoA (*47*), which also plays a role in endomitosis (*49*), we found that several centrosomal and cell cycle proteins accumulated upon proteasome inhibition, indicating that they would usually be degraded during proplatelet formation and might therefore be involved in its regulation (table S1). Collectively, our data suggest that proplatelet formation and the cell cycle may be intimately linked.

### Impaired proplatelet formation upon centrosome declustering in interphase

**Fig. 2.**
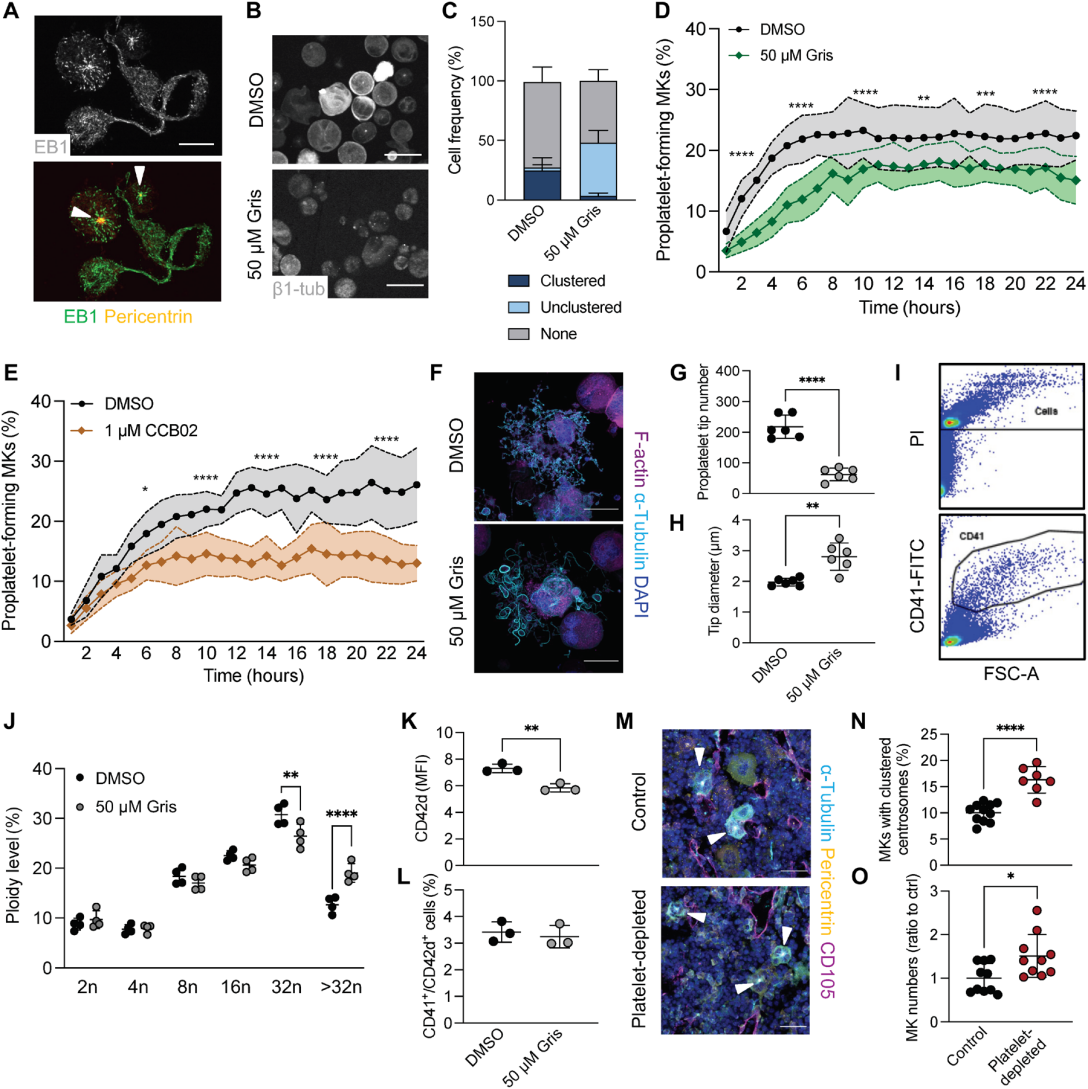
Centrosome clustering is essential for proplatelet formation and is enhanced during emergency thrombopoiesis. (**A**) Proplatelet-forming FLMKs were spun down onto coverslips and stained for EB3 and pericentrin. Scale bar, 30 μm. Arrowheads highlight clustered centrosomes. (**B** and **C**) Centrosome clustering was quantified in dimethyl sulfoxide (DMSO)–or Gris-treated FLMKs transduced with MSCV–β1-tubulin–dendra2. Images were acquired every hour for 24 hours. *n* = 3. At least 200 cells were analyzed per mouse. Data are presented as means ± SD. Scale bars, 10 μm. (**D** and **E**) Proplatelet formation of FLMKs treated with the interphase declustering drug Gris (D) or the CPAP inhibitor CCB02 (E) was assessed using the IncuCyte imaging system and a customized analysis pipeline (*87*). *n* = 3; four technical replicates. Data are presented as means ± SD. (**F**) BM MKs were treated with DMSO or 50 μM Gris and allowed to form proplatelets on a CD31-coated surface. MKs were stained for α-tubulin and F-actin. Nuclei were counterstained using 4′,6-diamidino-2-phenylindole (DAPI). Scale bars, 50 μm. (**G** and **H**) Proplatelet tip number and tip diameter were analyzed using ImageJ software. *n* = 2. Three representative cells were analyzed per mouse. Data are presented as means ± SD. Unpaired, two-tailed Student’s *t* test. **P < 0.01; ****P < 0.0001. FSC-A, forward scatter area. (**I** and **J**) Representative flow plot (I) and ploidy distribution (J) of DMSO- and Gris-treated, in vitro differentiated BM MKs were assessed using flow cytometry. Data are presented as means ± SD. Two-way ANOVA with Sidak correction for multiple comparisons. **P < 0.01; ****P < 0.0001. (**K** and **L**) CD42d MFI (K) and percentage of CD41/CD42d^+^ cells in DMSO- and Gris-treated cultures (L) were analyzed by flow cytometry. *n* = 4. Data are presented as means ± SD. Unpaired, two-tailed Student’s *t* test. **P < 0.01. (**M** to **O**) Representative images (M) and quantification of MKs containing clustered centrosomes (N) and MK numbers (O) in mice in situ upon platelet depletion. Samples were taken 96 hours after administration of an anti-GPIbα antibody. Scale bars, 50 μm. *n* = 10. Data are presented as means ± SD. Unpaired, two-tailed Student’s *t* test. *P < 0.05; ****P < 0.0001.

Our previous data suggested centrosome clustering coincided with the initiation of proplatelet formation. Immunofluorescent stainings for the microtubule-associated protein end-binding protein 1 (EB1) and the centrosomal protein pericentrin substantiated these findings by revealing that centrosome clustering exclusively occurred in nonproplatelet-forming MKs (Fig. 2A, white arrowheads). Furthermore, EB1-marked “comets” emanated from clustered centrosomes toward the cell periphery, providing evidence that supercentrosomes likely serve as MTOCs before proplatelet formation (movie S8) (*7*). To test whether inhibition of centrosome clustering would affect the maturation of MKs or their capacity to release proplatelets, we utilized the declustering agent griseofulvin (Gris), which causes cell death by mitotic catastrophe in cancer cells with clustered centrosomes (*50*, *51*). Treatment of FLMKs with Gris and subsequent live-cell imaging revealed fewer centrosome-containing MKs (Fig. 2, B and C), confirming that Gris induced centrosome declustering. In addition to the close connection previously described between centrosomes and the Golgi apparatus (*52*), we also observed a dispersion of Golgi ribbons upon Gris treatment (fig. S1, F and G).

To investigate the effect of centrosome declustering on proplatelet formation, FLMKs were next treated with Gris, and proplatelet formation was assessed using an in-house custom image analysis pipeline (*53*), which revealed a reduction in proplatelet formation upon centrosome declustering using 50 μM Gris (Fig. 2D and fig. S1H). Notably, proplatelet area was significantly reduced even at lower concentrations of Gris, suggesting impaired proplatelet elaboration and branching upon centrosome declustering (fig. S1I). Comparable findings were obtained with the declustering agent CCB02, which inhibits interactions of the centriolar protein centrosomal protein 4.1–associated protein (CPAP) with microtubules (Fig. 2E and fig. S1J). Immunostainings of proplatelet-forming MKs visualizing microtubules and F-actin further underlined a reduction in the number of proplatelet extensions upon Gris treatment (Fig. 2, F and G), suggesting aberrant microtubule redistribution in line with previously described inhibition of microtubule assembly (*7*). We also observed an increase in proplatelet size with higher α-tubulin intensity (Fig. 2H), strongly implying defective microtubule dynamics to account for the observed decrease in proplatelet area. Together, these data reveal an unknown role of centrosomes in the regulation of proplatelet formation.

To date, the contribution of centrosomes in MK maturation and endomitosis has not been investigated. Ploidy assessment revealed a reduction in 32n MKs upon Gris treatment, while the percentage of MKs with a higher ploidy was increased, suggesting that Gris enhanced polyploidization, potentially because MKs were unable to initiate proplatelet formation (Fig. 2J). While we did observe a slightly reduced mean fluorescence intensity (MFI) for the MK marker CD42d [glycoprotein V (GPV)] between vehicle- and Gris-treated cells (Fig. 2K), the percentage of CD41/CD42d^high^ cells was unaltered upon Gris treatment (Fig. 2L), suggesting that MKs were still able to mature upon inhibition of centrosome clustering.

We next extended our in vitro findings to explore the role of centrosome clustering in vivo. To model a setting of enhanced thrombopoiesis in which we expect an increased amount of MKs containing clustered centrosomes, we mimicked thrombocytopenia using a murine model of glycoprotein Ibα (GPIbα)-mediated platelet depletion. Femoral cryosections of untreated animals revealed centrosome clustering in situ comparable to our observations in vitro (Fig. 2M and fig. S1K). Similarly, MKs with clustered centrosomes in situ exhibited a higher nuclear-to-cytoplasmic ratio (fig. S1L), suggesting that they represent a distinct subset of MKs highly dependent on centrosomal dynamics. We observed an increased number of supercentrosome-containing MKs during rebound thrombopoiesis 96 hours after depletion in conjunction with mildly enhanced MK numbers (Fig. 2, N and O), suggesting that thrombocytopenia-induced platelet production was accompanied by centrosome clustering. However, whether this increase simply reflects a general adaptation of different MK subsets to platelet needs remains to be determined. In summary, our data demonstrate that inhibition of centrosome clustering impairs proplatelet formation and that clustered centrosomes are responsive to acute platelet production.

### Enforced G_1_ cell cycle arrest enhances proplatelet formation

**Fig. 3.**
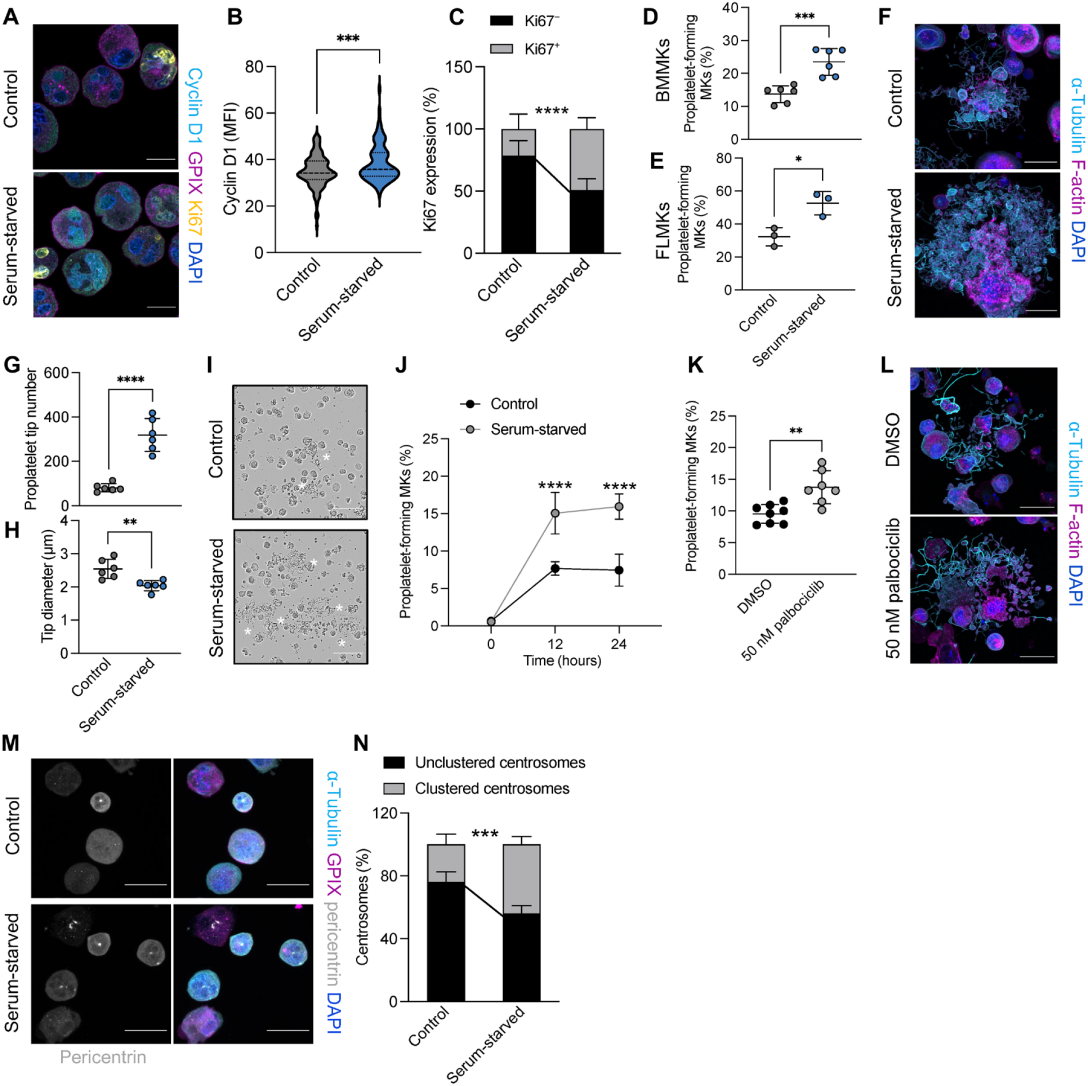
G_1_ cell cycle arrest increases proplatelet formation. (**A**) Visualization of cyclin D1 and the cell cycle marker Ki67 in BM MKs incubated in control or serum-free medium for 3 hours. Scale bars, 20 μm. (**B** and **C**) Quantification of cyclin D1 MFI (B) and Ki67 expression (C) of cells shown in (A). Data are presented as means ± SD. *n* = 3. Unpaired, two-tailed Student’s *t* test. ***P < 0.001; ****P < 0.0001. (**D** and **E**) Proplatelet formation from control and serum-starved BM MKs (D) and FLMKs (E) was quantified after 24 hours using an automated imager. Data are presented as means ± SD. *n* = 6 (D) and *n* = 3 (E). Unpaired, two-tailed Student’s *t* test. *P < 0.01; ***P < 0.0001. (**F**) BM MKs were incubated in control or serum-free medium and allowed to form proplatelets on a CD31-coated surface. MKs were stained for α-tubulin and F-actin. Nuclei were counterstained using DAPI. Scale bars, 25 μm. (**G** and **H**) Proplatelet tip number and tip diameter were analyzed using ImageJ software. *n* = 6. Data are presented as means ± SD. Unpaired, two-tailed Student’s *t* test. **P < 0.01; ****P < 0.0001. (**I** and **J**) Visualization and quantification of proplatelet formation of BM MKs in control and serum-free medium were assessed using the IncuCyte imaging system and a customized analysis pipeline. *n* = 4; four technical replicates. Data are presented as means ± SD. Two-way ANOVA with Sidak correction for multiple comparisons. ***P < 0.001. Scale bar, 100 µm. (**K**) Proplatelet formation of BM MKs treated with DMSO or 50 nM palbociclib for 24 hours was quantified using an automated imager. Data are presented as means ± SD. *n* = 8. Unpaired, two-tailed Student’s *t* test. **P < 0.01. (**L**) Representative images of BM MKs treated with DMSO or 50 nM palbociclib forming proplatelets on a CD31-coated surface. MKs were stained for α-tubulin and F-actin. Nuclei were counterstained using DAPI. Scale bars, 25 μm. (**M**) Control and serum-starved MKs were fixed and stained for α-tubulin, GPIX, and pericentrin. Scale bars, 50 μm. (**N**) The percentage of MKs containing clustered centrosomes was quantified manually using ImageJ software. *n* = 4. Data are presented as means ± SD. Two-way ANOVA with Sidak correction for multiple comparisons. ***P < 0.001.

Our findings suggest that in addition to regulating centrosomal clustering, the cell cycle state of MKs likely governs their ability to produce platelets. Since FUCCI-transduced MKs exclusively generated platelets in G_1_ (see Fig. 1F), we investigated whether G_1_ cell cycle arrest affected proplatelet formation rates. Cell cycle synchronization can be initiated via different mechanisms: (i) serum starvation (*54*), which induces cell cycle arrest in G_1_; or (ii) treatment with cell cycle agents such as palbociclib (*42*), which inhibit cyclin-dependent kinase 4 (Cdk4) and Cdk6 and thus prevent G_1_-S transition. To verify that cell cycle arrest was achieved during serum starvation, we incubated BM MKs in serum-free medium for 3 hours and visualized cyclin D1 expression, which is restricted to cells in G_1_, and Ki67, a marker of cells actively undergoing the cell cycle (*55*). Cyclin D1 expression was significantly higher in MKs following serum starvation (Fig. 3, A and B), which was accompanied by a marked increase in the number of MKs positive for Ki67 (Fig. 3C), thus confirming cell cycle initiation but arrest in G_1_ in MKs following serum starvation.

Next, BM MKs and FLMKs were incubated in serum-free medium for 24 hours, during which we observed a significant increase in the number of proplatelet-forming MKs (Fig. 3, D and E). Analysis of proplatelet-forming MKs revealed highly elaborate proplatelet structures after serum starvation with increased proplatelet tips (Fig. 3, F and G), albeit a smaller diameter (Fig. 3H). We also observed a distinct increase in proplatelet formation when BM MKs were incubated in serum-free medium without hirudin (Fig. 3, I and J), which is usually essential for BM MKs to generate proplatelets in vitro (*56*). Proplatelet formation and elaboration were similarly increased when BM MKs were treated with the Cdk4/6 inhibitor palbociclib (Fig. 3, K and L). In line with our hypothesis that centrosome clustering preceded proplatelet formation, we also observed a significant increase in supercentrosome-containing MKs after serum starvation for 4 hours before proplatelet formation (Fig. 3, M and N). In summary, these findings support our hypothesis that proplatelet formation is cell cycle dependent and can be promoted via cell cycle arrest in G_1_ in vitro.

### STS in mice increases platelet production

**Fig. 4.**
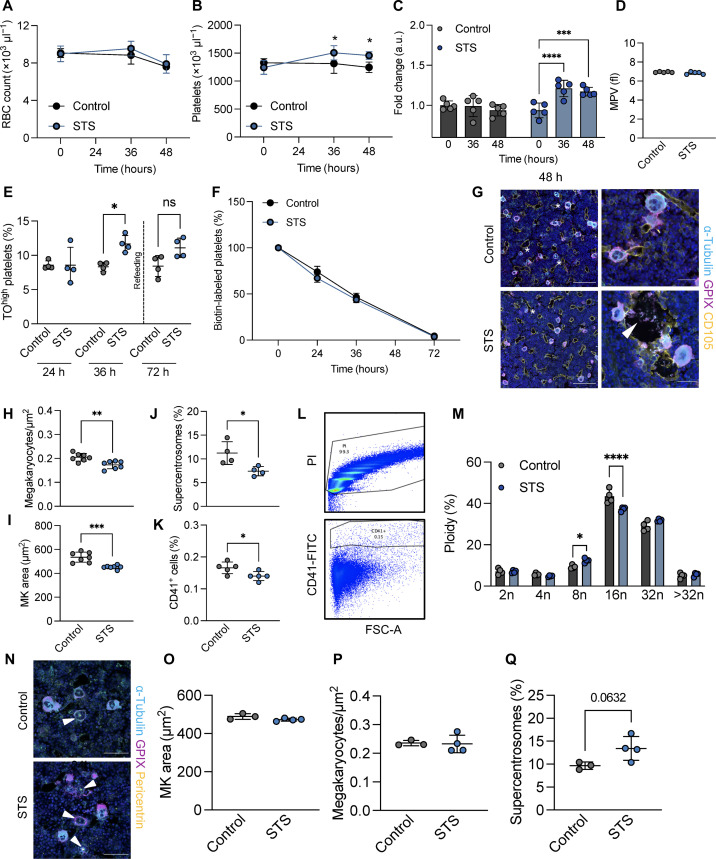
STS increases proplatelet formation in vivo. (**A** and **B**) Red blood cell (A) and platelet counts (B) in control and STS mice were analyzed after 36 and 48 hours. *n* = 5. Data are presented as means ± SD. Two-way ANOVA with Sidak correction for multiple comparisons. *P < 0.05. (**C**) Platelet count fold change in control and STS mice. *n* = 5. Data are presented as means ± SD. Two-way ANOVA with Sidak correction for multiple comparisons. ***P < 0.001; ****P < 0.0001. (**D**) MPV was assessed at an automated blood cell analyzer. *n* = 5. Data are presented as means ± SD. (**E**) The percentage of thiazole orange (TO)^high^ platelets over time was analyzed by flow cytometry. Dashed line represents the time of refeeding. *n* = 4. Data are presented as means ± SD. Unpaired, two-tailed Student’s *t* test. *P < 0.05. ns, not significant. (**F**) The percentage of biotin-labeled platelets over time in control and STS mice was assessed by flow cytometry. Data are presented as means ± SD. *n* = 4. (**G**) Femoral cryosections of control and STS mice (48 hours) were stained for GPIX, CD105, and α-tubulin. Stars highlight MKs containing clustered centrosomes. White arrowhead points to platelets being released into sinusoids. Scale bars, 100 μm and 30 μm (zoom-ins). (**H** and **I**) MK area and numbers in femoral cryosections derived from control and STS mice were quantified using automated image analysis software. *n* = 5. Data are presented as means ± SD. Unpaired, two-tailed Student’s *t* test. **P < 0.01; ***P < 0.001. (**J**) The number of MKs containing clustered centrosomes was analyzed in femoral cryosections. *n* = 5. Data are presented as means ± SD. Unpaired, two-tailed Student’s *t* test. *P < 0.05. (**K** to **M**) Numbers of CD41-positive cells (K), representative flow plot (L), and ploidy distribution (M) were analyzed by flow cytometry. *n* = 5. Data are presented as means ± SD. Two-way ANOVA with Sidak correction for multiple comparisons. *P < 0.05; ****P < 0.0001. (**N**) Femoral cryosections of control and STS mice (24 hours) were stained for GPIX, pericentrin and α-tubulin. Arrowheads highlight MKs containing clustered centrosomes. Scale bars, 100 μm. (**O** and **P**) MK area and numbers in femoral cryosections derived from control and STS mice were quantified using automated image analysis software. *n* = 5. Data are presented as means ± SD. Unpaired, two-tailed Student’s *t* test. (**Q**) The number of MKs containing clustered centrosomes was analyzed in femoral cryosections. *n* = 5. Data are presented as means ± SD. Unpaired, two-tailed Student’s *t* test.

To date, therapeutics that increase platelet counts in patients rely on enhancing MK numbers, commonly in response to TPO mimetics such as eltrombopag or romiplostim (*57*), which can take days to weeks for maximum efficacy. In contrast, immediately boosting platelet counts by increasing platelet production from existing MKs has rarely been therapeutically investigated because of a lack of knowledge about what initiates platelet release. To assess whether the increased proplatelet formation we observed upon serum starvation in vitro had in vivo relevance, we performed STS experiments in mice and assessed platelet counts after 0, 36, and 48 hours of food deprivation. As expected, STS reduced body weight beginning at 24 hours (fig. S2A). While we observed a significant decrease in white blood cell counts, as previously described (*58*), which was mostly due to lymphocyte egress (fig. S2C), red blood cell counts remained largely unaltered (Fig. 4A). Platelet counts, however, significantly increased in STS mice from 36 hours onward (Fig. 4, B and C). Although mean platelet volume (MPV) was unaltered (Fig. 4D), we observed a slight reduction in CD42b expression (fig. S2D).

To test whether the increase in platelet counts reflected an increase in platelet production, we assessed the amount of reticulated, thiazole orange (TO)^high^ platelets, which is a reliable measure of platelet age (*59*). While no difference was detectible after 24 hours of STS, the proportion of TO^high^ platelets was significantly increased after 36 hours of STS (Fig. 4E), in line with the increased platelet count at that time point. After refeeding, however, the amount of TO^high^ platelets gradually reverted back to baseline. To further verify that platelets were newly generated upon STS, we intravenously injected control and STS mice with biotin, which labels intravascular cells including platelets and can be tracked over time. Consistent with the increase in TO^high^ platelets, no difference in platelet clearance was observed during STS (Fig. 4F), strongly suggesting that increased platelet counts were due to enhanced platelet production from existing MKs. To further exclude that platelet counts upon STS were increased because of reduced platelet clearance, we assessed the spleens of control and STS mice. Spleens were slightly smaller in STS mice, although not significantly when normalized to body mass (fig. S2E), and splenic CD41 MFI as a measure for platelets entrapped in the splenic red pulp was unaltered (fig. S2, F and G), suggesting that elevated platelet counts were not due to decreased platelet turnover. Splenic MK numbers were also comparable between control and STS mice (fig. S2H). While platelet degranulation assessed by surface P-selectin exposure was unaltered (fig. S2I), platelets from STS mice were partially sensitized to integrin α_IIb_β_3_ activation following the GPVI agonist cross-linked collagen-related peptide (CRP-XL) (fig. S2J), which was previously described as a feature of newly generated platelets (*60*).

We next assessed the effect of STS on the BM. MKs in femoral cryosections demonstrated a notable amount of platelet release events into vessel sinusoids (Fig. 4G). The overall number of MKs was decreased with a concomitant reduction in their area, suggesting that larger MKs had released platelets (Fig. 4, H and I). Notably, the number of MKs containing clustered centrosomes was also slightly lower upon STS, suggesting that clustering preceded platelet production (Fig. 4J). The decrease in MKs was also evident by flow cytometry (Fig. 4K). STS mice further exhibited a slight increase in lower ploidy 8n MKs, while 16n MKs were markedly reduced (Fig. 4, L and M). To test whether centrosome clustering preceded platelet production during STS, we also analyzed femoral BM sections after 24 hours of STS and found no difference in either MK size (Fig. 4, N and O) or count (Fig. 4P). Moreover and consistent with our data at 48 hours, we observed a trending increase in the number of MKs containing supercentrosomes at 24 hours (Fig. 4Q), suggesting that MKs clustered their centrosomes before platelet release.

Recent data have shown that stimulation of the glucocorticoid receptor in MKs can induce proplatelet formation in vitro and in vivo (*61*), and cortisol levels were shown to spike upon food deprivation (*62*). To assess the role of cortisol in our STS model, we measured cortisol levels in the blood plasma of control and STS mice and only observed a tendency toward increased cortisol levels upon STS (fig. S2K), suggesting that alternative mechanisms (such as cell cycle arrest) were responsible for the increase in platelet production. In line with the increased platelet counts, TPO levels were mildly reduced upon STS (fig. S2L). We did not detect overt differences in cytokines present in BM supernatant or blood plasma (fig. S2, M and N). In summary, our data reveal a cell cycle–driven mechanism that coordinates supercentrosome formation to regulate proplatelet formation from murine MKs, which may open avenues for therapeutically targeting MKs.

### Centrosome clustering in MKs is dependent on kinesin-14 family member KIFC1

**Fig. 5.**
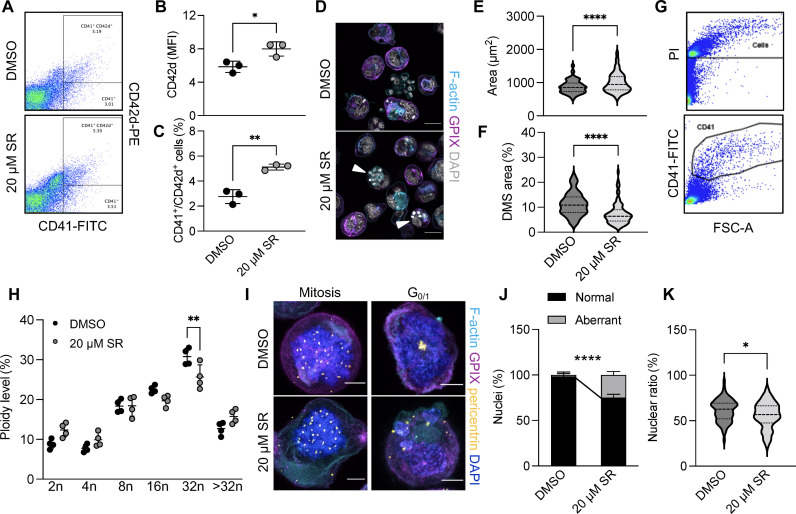
KIFC1 is essential for centrosome clustering in murine MKs. (**A** to **C**) Representative flow plots (A) and quantification of CD42d MFI (B) and CD41/CD42d-positive cells (C) of DMSO- and SR-treated, in vitro differentiated BM MKs. *n* = 3. Data are presented as means ± SD. Unpaired, two-tailed Student’s *t* test. *P < 0.05; **P < 0.01. (**D**) Visualization of the DMS (GPIX and Phalloidin) in BM MKs treated with DMSO or 20 μM SR after 4 days in culture. Scale bars, 25 μm. (**E** and **F**) Quantification of cell size (E) and DMS area in relation to cell size (F) in MKs treated with DMSO or 20 μM SR. *n* = 3. Data are presented as means ± SD. Unpaired, two-tailed Student’s *t* test. ****P < 0.0001. (**G** and **H**) Ploidy distribution of DMSO- and SR-treated, in vitro differentiated BM MKs was assessed using flow cytometry. *n* = 4. Data are presented as means ± SD. Two-way ANOVA with Sidak correction for multiple comparisons. **P < 0.01. (**I**) Visualization of DNA and centrosomes in BM MKs treated with DMSO or 20 μM SR undergoing different cell cycle stages. Scale bars, 10 μm. (**J** and **K**) Quantification of micronucleus formation (J) and nuclear ratio in relation to cell size (K) in BM MKs treated with DMSO or 20 μM SR. Data are presented as means ± SD. Unpaired, two-tailed Student’s *t* test. *P < 0.05; ****P < 0.0001.

We next aimed to determine the mechanism driving centrosome clustering in MKs. The kinesin-14 family member KIFC1 is essential for centrosome clustering in cancer cells, and treatment of cells containing supernumerary centrosomes with a KIFC1 inhibitor results in cell death due to mitotic catastrophe (*36*, *63*). We thus hypothesized that KIFC1 may similarly contribute to centrosome clustering in MKs. First, we assessed whether inhibition of KIFC1-mediated centrosome clustering affected MK differentiation and polyploidization. To this end, we treated lineage-depleted hematopoietic progenitor cells with dimethyl sulfoxide (DMSO) or 20 μM of an established inhibitor of KIFC1, SR31527 (SR) (*29*, *35*), on day 0 and cultured them in the presence of TPO. On day 3, we assessed the surface expression of CD41 and CD42d by flow cytometry (Fig. 5A). We observed both an increase in the MFI for CD42d and a higher number of CD41/CD42d^high^ cells (Fig. 5, B and C). In contrast, despite an increase in cell size (Fig. 5, D and E) that correlated to the increased CD41/CD42d surface expression, immunofluorescence stainings for the DMS markers CD42a (GPIX) and F-actin revealed a significant reduction in DMS area (Fig. 5F). In addition, SR did not affect polyploidization (Fig. 4, I and J) except for a minor shift from 16 to 32n MKs. A previous study by Trakala *et al.* (*26*) identified that MKs can switch their endomitotic cycles to endoreplication upon inhibition of mitosis, which would not necessarily be apparent when analyzing ploidy by flow cytometry. We therefore visualized centrosomes and DNA in MKs treated with DMSO or SR after 4 days of culturing during different cell cycle stages. While SR-treated MKs were able to form multipolar spindles during mitosis, we observed the formation of aberrant nuclear structures including micronuclei (Fig. 5, I and J), as previously suggested (*41*). Furthermore, SR treatment led to an overall reduction in the nuclear to cytoplasmic ratio (Fig. 5K), likely caused by altered endomitosis, thus linking centrosome clustering to cell cycle progression during MK maturation.

### Reduced proplatelet formation upon KIFC1 inhibition

**Fig. 6.**
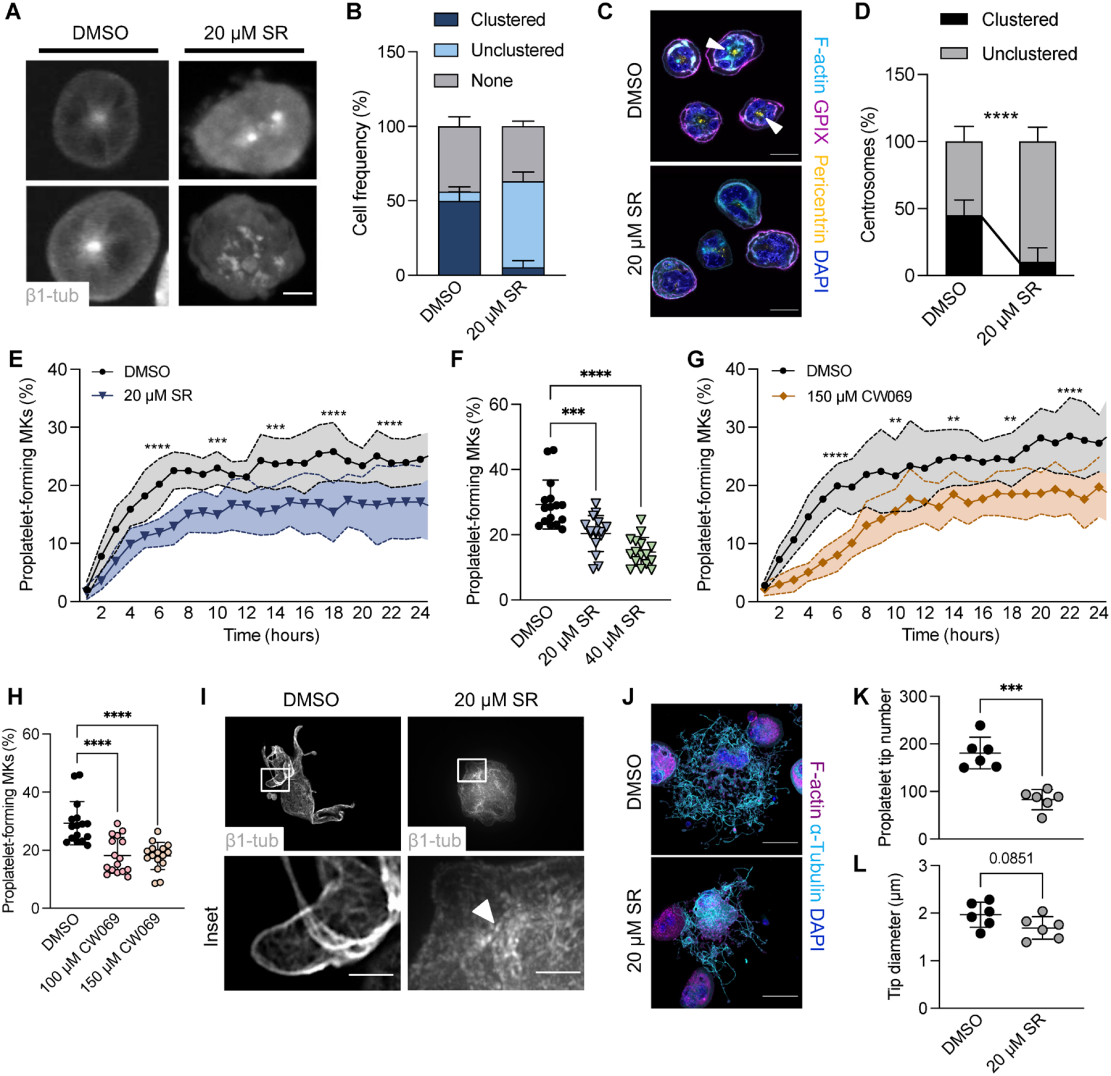
Impaired proplatelet formation following KIFC1 inhibition in murine MKs in vitro. (**A** and **B**) Visualization (A) and quantification (B) of clustered centrosomes in FLMKs by live-cell imaging treated with DMSO or 20 μM KIFC1 inhibitor SR. Image acquisition was performed every hour. Scale bar, 10 μm. At least 100 cells were analyzed per mouse. Data are presented as means ± SD. (**C** and **D**) Visualization (C) and quantification (D) of clustered centrosomes in BM MKs treated with DMSO or the KIFC1 inhibitor SR (20 μM). *n* = 2. Scale bars, 25 μm. At least 100 cells were analyzed per mouse. Data are presented as means ± SD. Unpaired, two-tailed Student’s *t* test. ****P < 0.0001. (**E**) Proplatelet formation of FLMKs treated with DMSO or 20 μM SR was assessed using the IncuCyte imaging system and a customized analysis pipeline. *n* = 4; four technical replicates. Data are presented as means ± SD. Two-way ANOVA with Sidak correction for multiple comparisons. ***P < 0.001; ****P < 0.0001. (**F**) Percentage of proplatelet-forming MKs at 24 hours upon treatment with DMSO or 20 or 40 μM SR. *n* = 4; four technical replicates. Data are presented as means ± SD. One-way ANOVA with Sidak correction for multiple comparisons. ***P < 0.001; ****P < 0.0001. (**G**) Proplatelet formation from FLMKs upon treatment with DMSO or 150 μM CW069 was analyzed using the IncuCyte imaging system and a custom analysis pipeline. *n* = 4; four technical replicates. Data are presented as means ± SD. Two-way ANOVA with Sidak correction for multiple comparisons. **P < 0.01; ****P < 0.0001. (**H**) Percentage of proplatelet-forming MKs at 24 hours upon treatment with DMSO or 100 or 150 μM CW069. Data are presented as means ± SD. One-way ANOVA with Sidak correction for multiple comparisons. ****P < 0.0001. (**I**) DMSO- and SR-treated, MSCV–β1-tubulin–dendra2–transduced FLMKs were spun down onto coverslips. Scale bars, 20 μm and 5 μm (inset). (**J**) BM MKs were treated with DMSO or 20 μM SR and were allowed to form proplatelets on a CD31-coated surface for 24 hours. MKs were stained for α-tubulin and F-actin. Scale bars, 50 μm. (**K** and **L**) Proplatelet tip number and tip diameter were analyzed using ImageJ software. *n* = 6. Data are presented as means ± SD. Unpaired, two-tailed Student’s *t* test. ***P < 0.001.

In addition to its newly recognized role during MK maturation and endomitosis, our previous data suggest that centrosome clustering precedes proplatelet formation. To first validate centrosome declustering upon KIFC1 inhibition, we treated FLMKs before proplatelet formation with the KIFC1 inhibitor SR for 2 hours and observed that only 6% of round MKs contained clustered centrosomes compared to 50% in control cells, strongly suggesting that KIFC1 mediated centrosome clustering in MKs (Fig. 6, A and B). We confirmed these findings in BM MKs, in which, virtually, all supercentrosomes were declustered 3 hours after the addition of SR (Fig. 6, C and D). We next examined whether inhibition of KIFC1-dependent centrosome clustering affected proplatelet formation. Treatment of FLMKs with SR led to a decrease in, albeit not completely abolished, proplatelet formation (Fig. 6, E and F, and movies S9 and S10). Similarly to SR, another KIFC1 inhibitor, CW069 (*37*), inhibited proplatelet formation to a similar extent (Fig. 6, G and H). Critically, treatment with either of the inhibitors used in this study did not result in significant levels of cytotoxicity on either progenitors or mature MKs, respectively (fig. S3, A and B). In contrast to the vehicle control, we did not observe microtubule relocalization to the cell cortex in SR-treated MKs (Fig. 6I, white arrowhead). Immunostainings for F-actin and α-tubulin further revealed broad alterations in proplatelet morphology (Fig. 6J), including less branching of proplatelets upon SR treatment, a reduction in the number of proplatelet tips, and aberrant platelet sizing (Fig. 6, K and L). Overall, these findings suggest an important role of KIFC1 in regulating centrosome clustering before proplatelet formation.

Our experimental findings can further be supported by computational modeling of two recent studies (fig. S3C) (*64*, *65*). Kinesin-14 motors such as KIFC1 at the overlap between microtubules generate intercentrosomal attraction forces (fig. S3C1). Centrosomes are attracted to chromosomes via dynein motors. Microtubule^+^ ends growing into the chromosome arms interact with chromokinesin motors, which push the microtubule^+^ ends away from the chromosomes causing the effective repulsion force between the centrosome and chromosome (fig. S3C2) (*64*, *65*). The attraction between the centrosomes causes their partial clustering, but the chromosomes at the center block the small centrosomal clusters from being bridged by microtubules and keep the clusters too far apart for KIFC1 to be effective, thus explaining why we observe multipolar spindles in the presence of chromosomes. After DNA decondensation, the dominant KIFC1-mediated attraction collapses all centrosomal clusters into one cluster (fig. S3, C3 and C4), which is why the monopolarity is suppressed as soon as KIFC1 is inhibited. The cross-linking action of microtubule-associated proteins tends to increase with microtubule density, which may cause enhanced and accelerated microtubule bundling in monopolar cells (fig. S3C4), resulting in thicker microtubule bundles, which are more mechanically sturdy and may explain the extensive formation of microtubule-rich protrusions from MKs.

### KIFC1-deficient mice exhibit reduced platelet counts despite increased MK numbers

**Fig. 7.**
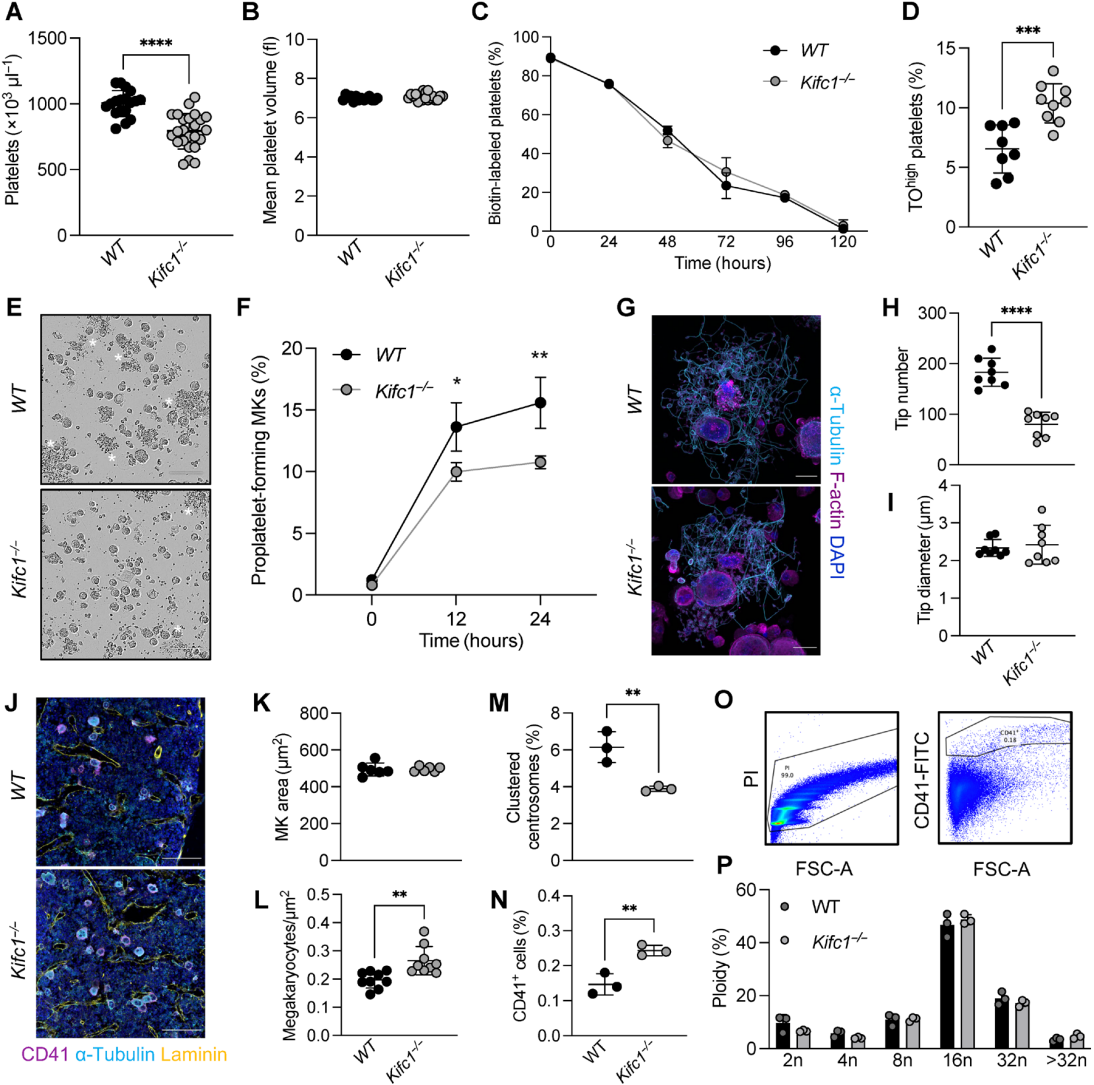
*Kifc1*^−/−^ mice display an isolated reduction in platelet counts despite increased MK numbers. (**A** and **B**) Platelet count and size in WT and *Kifc1*^−/−^ mice were assessed using an automated blood cell analyzer. *n* = 25. Data are presented as means ± SD. Unpaired, two-tailed Student’s *t* test. ****P < 0.0001. (**C**) The percentage of biotin-labeled platelets over time in WT and *Kifc1*^−/−^ mice was assessed by flow cytometry. Data are presented as means ± SD. *n* = 3. (**D**) The percentage of TO^high^ platelets derived from WT and *Kifc1*^−/−^ mice was assessed by flow cytometry. *n* = 8. Data are presented as means ± SD. Unpaired, two-tailed Student’s *t* test. ***P < 0.001. (**E** and **F**) Proplatelet formation from BM MKs derived from WT and *Kifc1*^−/−^ mice was analyzed using the IncuCyte imaging system and quantified manually. *n* = 3; four technical replicates. Data are presented as means ± SD. Two-way ANOVA with Sidak correction for multiple comparisons. *P < 0.05; **P < 0.01. (**G**) Proplatelet-forming MKs from WT or *Kifc1*^−/−^ mice were stained for α-tubulin and F-actin and visualized by confocal microscopy. Scale bars, 30 μm. (**H** and **I**) Proplatelet tip number and tip diameter were analyzed using ImageJ software (National Institutes of Health). *n* = 8. Data are presented as means ± SD. Unpaired, two-tailed Student’s *t* test. ****P < 0.0001. (**J**) Femoral cryosections of WT and *Kifc1*^−/−^ mice were stained for CD41, laminin, and α-tubulin. Scale bars, 100 μm. (**K** to **M**) MK area, numbers, and MKs containing clustered centrosomes in femoral cryosections derived from WT and *Kifc1*^−/−^ mice were quantified using an automated image analysis software. *n* = 6. Data are presented as means ± SD. Unpaired, two-tailed Student’s *t* test. **P < 0.01. (**N** to **P**) Numbers of CD41-positive cells (N) and ploidy distribution [(O) and (P)] were analyzed by flow cytometry. *n* = 3. Data are presented as means ± SD. Two-way ANOVA with Sidak correction for multiple comparisons. **P < 0.01.

To validate our in vitro findings, we next investigated whether inhibition of centrosome clustering resulted in altered platelet production in vivo. We acquired constitutive KIFC1-deficient (*Kifc1*^−/−^) mice, previously generated in the Knockout Mouse Project, and validated the deletion of the *Kifc1* gene by polymerase chain reaction (PCR) (fig. S4A). Litter sizes of *Kifc1*^−/−^ mice were slightly lower (fig. S4B), but we observed no difference in the ratio between male and female mice born to *Kifc1*^−/−^ mice when compared to an internal wild-type (WT) control (fig. S4C). While red blood cell counts were unaltered (fig. S4D) and white blood cell counts were mildly, albeit not statistically significantly, increased (fig. S4E), lack of KIFC1 resulted in a 20% reduction in platelet counts (1034 ± 83 in WT versus 856 ± 93 in *Kifc1*^−/−^ mice) but unaltered platelet size (Fig. 7, A and B). To test whether enhanced platelet clearance accounted for the decrease in platelet counts, we assessed the proportion of biotin-positive platelets in WT and *Kifc1*^−/−^ mice following intravenous biotin administration and found no differences in platelet clearance over 5 days (Fig. 7C), suggesting impaired production accounts for the reduction in platelet counts. At the same time, however, we did not observe differences in platelet rebound between WT and *Kifc1*^−/−^ mice, when assessing platelet recovery upon depletion using a GPIbα antibody (fig. S4F). To probe these results further, we next assessed the amount of reticulated platelets in WT and *Kifc1*^−/−^ mice. We found that both immature platelet fraction (fig. S4G) and the number of TO^high^ platelets (Fig. 7D) were increased in *Kifc1*^−/−^ mice. Platelet α_IIb_β_3_ integrin activation and α-granule degranulation following stimulation with various agonists, however, were mostly unaltered between platelets derived from WT or *Kifc1*^−/−^ mice (fig. S4, H and I), except for a mild hyperreactivity toward low-dose CRP-XL stimulation.

To further delineate how the lack of KIFC1 affected megakaryopoiesis and thrombopoiesis, we next investigated proplatelet formation from cultured BM MKs. MKs derived from *Kifc1*^−/−^ mice exhibited reduced proplatelet formation compared to WT controls (Fig. 7, E and F, white asterisks). Moreover, immunofluorescence stainings revealed a reduced number of proplatelet tips in *Kifc1*^−/−^ MKs (Fig. 7, G and H), which was in line with the aberrant proplatelets formed upon KIFC1 inhibition (see Fig. 6J). The proplatelet tip diameter, however, was unaltered (Fig. 7I). Maturation of *Kifc1*^−/−^ MKs in vitro as assessed by flow cytometry was unaffected (fig. S4J). Notably, in vitro matured *Kifc1*^−/−^ MKs contained a reduced but not abolished number of clustered centrosomes (fig. S4K), suggesting that, despite the clear effects of pharmacological KIFC1 inhibition on centrosome clustering (see Fig. 6, A and B), other mechanisms or pathways compensated for the lack of KIFC1 in *Kifc1*^−/−^ mice. Regardless of the decrease in platelet counts, TPO levels in the blood plasma of *Kifc1*^−/−^ mice were unaltered (fig. S4L), which prompted us to assess MKs in femoral cryosections. MK area was unaltered (Fig. 7, J and K), while MK numbers were increased in *Kifc1*^−/−^ mice (Fig. 7L), further implying compensation of KIFC1 function through up-regulation of megakaryopoiesis. This coincided with a reduced number of MKs containings clustered centrosomes in situ (Fig. 7M). The increase in MK numbers was further verified by flow cytometry (Fig. 7N), underscoring the deficit in platelets produced per MK lacking KIFC1. Notably, ploidy was not significantly altered (Fig. 7, O and P). In summary, we have identified a role for the centrosomal protein KIFC1 in regulating centrosomal clustering in MKs, inhibition of which hampers platelet production both in vitro and in vivo.

## DISCUSSION

**Fig. 8.**
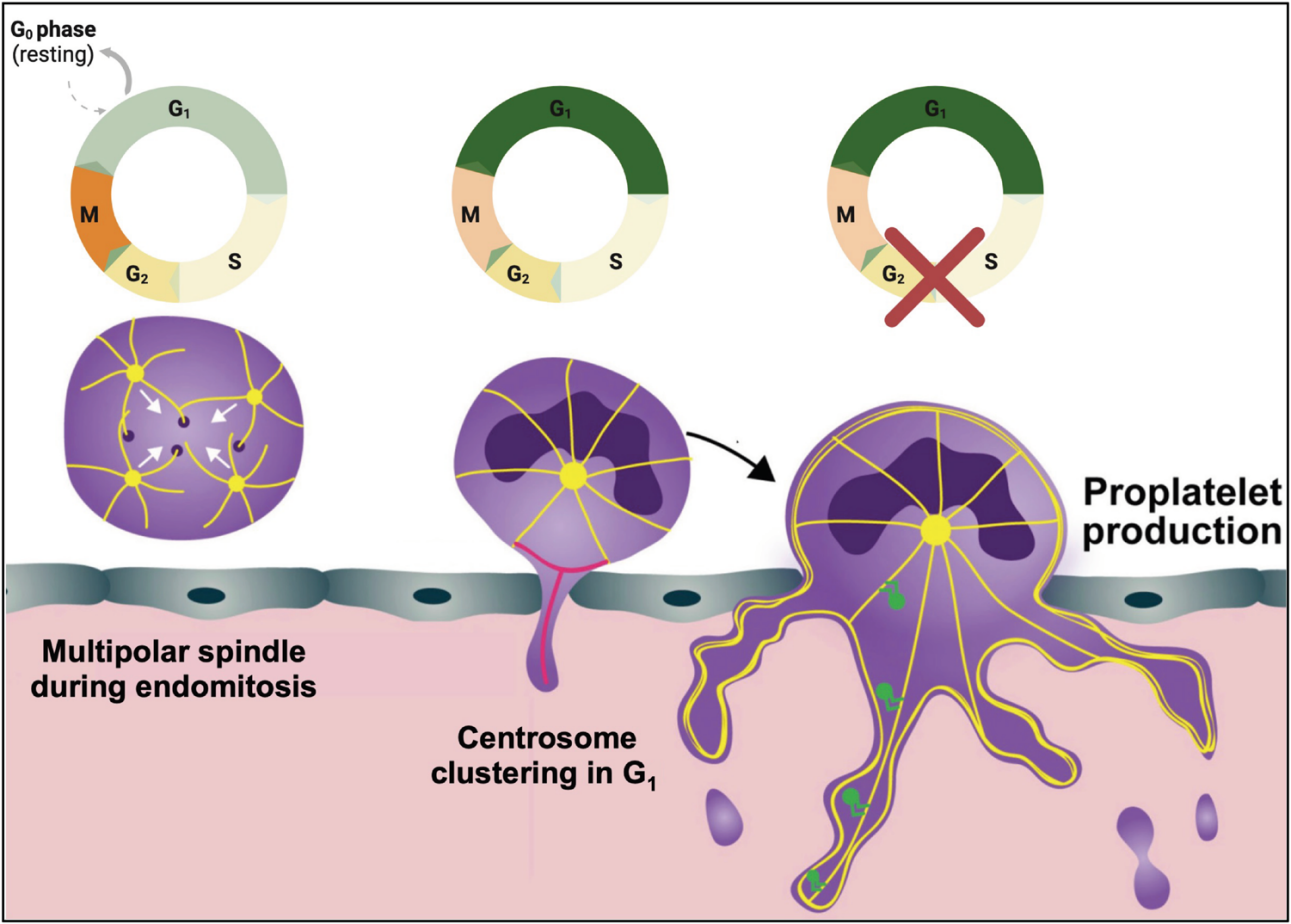
Summary schematic. MKs in the BM can form multipolar spindles during maturation and instead cluster their supernumerary centrosomes following mitosis. This clustering is cell cycle dependent and first appears in G_1_ but can last through S-G_1_ phase. Platelet production exclusively occurs in G_1_ and can be induced by cell cycle arrest, accompanied by increased centrosome clustering. Image was created using Biorender.com.

Because of the high demand for platelet transfusions and the short shelf-life of platelet donation concentrates, an important clinical application for MK research is to generate platelets in vitro from human induced pluripotent stem cells. While several models yield promising preliminary results (*66*–*69*), one major obstacle remains the synchronization of platelet production, since the triggers that induce platelet production remain unknown. Similar obstacles remain in vivo, where therapeutical interventions conventionally aim to increase MK numbers (*57*), rather than directly boosting platelet production from existing MKs. In this study, we provide the first report of centrosome clustering in hematopoietic cells and evidence that it plays a critical role in proplatelet formation, likely serving as an MTOC for microtubule reorganization (Fig. 8). Further, we identify KIFC1 as a centrosomal motor protein that promotes clustering of centrosomes in MKs both in vitro and in vivo.

Microtubules are essential for platelet production, and both the expression of specific microtubule subunits (*70*, *71*) and microtubule-regulating proteins (*72*) and posttranslational modifications on microtubules (*9*) have been described to govern proplatelet formation. However, despite their role as MTOCs, whether centrosomes might regulate microtubule dynamics during proplatelet formation has not been investigated to date. In nonmalignant cells, centrosomes duplicate once during each cell cycle and serve the formation of bipolar spindle poles for chromosomal segregation (*31*, *73*). The formation of these clustered centrosomes has therefore mostly been described as a mechanism in cancer cells to avoid aneuploidy, inhibition of which can result in cancer cell death (*31*, *50*, *74*). The up-regulation of KIFC1 in a variety of cancer cells with supernumerary centrosomes led researchers to develop small molecules specifically targeting KIFC1-dependent centrosomal clustering (*40*, *63*, *74*). Recent findings suggest that a small number of other cell types are also able to obtain supernumerary centrosomes and can cluster them into MTOCs, which, for example, enables F-actin ring formation in osteoclasts or increases locomotion of dendritic cells (*33*, *34*). Our study reveals that centrosomal clustering appears to serve very distinct, nonoverlapping purposes in cancer cells and MKs. Centrosome clustering in MKs occurred in G_1_ of interphase, instead of serving the formation of a bipolar spindle as observed in cancer cells. However, as in cancer cells, it was also dependent on KIFC1, inhibition of which reduced centrosomal clustering and subsequent proplatelet formation (Fig. 6). Our data suggest that centrosome clustering occurs before proplatelet formation and, in contrast to cancer cells, its inhibition only mildly affected MK maturation, thus implying that centrosome components were predominantly important for proplatelet initiation. Nonetheless, similar to previous studies (*41*), we identified that KIFC1 inhibition affected cell cycle dynamics during maturation, leading to micronucleus formation and defective endomitosis. Moreover, MKs containing clustered centrosomes had a higher nuclear-to-cytoplasmic ratio, suggesting that centrosome clustering relates to polyploidization and DMS formation.

The marginal reduction in platelet counts, despite an increase in TO^high^ platelets and increased MK numbers in the BM (Fig. 7) observed in *Kifc1*^−/−^ mice, might be due to several reasons: (i) As mentioned above, a variety of other molecules are important for centrosome clustering and might compensate for the lack of KIFC1; (ii) centrosome clustering may only be critical in a subset of MKs, and platelet production might occur via different mechanisms (*12*, *14*); or (iii) *Kifc1*^−/−^ mice mimic emergency megakaryopoiesis and thrombopoiesis leading to enhanced but atypical production to keep up with platelet demand. Further studies are needed to identify the underlying mechanisms in more detail. Notably, since centrosome clustering appears to be selectively important for cells containing numerous centrosomes, inhibition of KIFC1 would most likely affect not only cancer cells but also other polyploid cells such as MKs, which should be taken into account for potential future clinical approaches of KIFC1 inhibitors, as they may result in altered megakaryopoiesis and thrombocytopenia as a side effect.

Previous studies have demonstrated that cell cycle velocities affect hematopoietic cell fate decisions in MK-erythroid progenitors, with slower cell cycle speeds leading to an MK bias (*42*). In addition, past reports show that deletion of cell cycle regulators affects megakaryopoiesis and endomitosis (*26*); however, whether the cell cycle directs proplatelet formation had not been investigated. Our data using serum starvation or Cdk4/6 inhibition (Fig. 3) suggest that enforcing MKs to remain in G_1_ is sufficient to induce both centrosome clustering and subsequent proplatelet formation. These findings propose an important cell cycle dependence of proplatelet formation from MKs, which we further validated in vivo using a starvation model, revealing a direct production of platelets from existing MKs during STS (Fig. 4). Important recent data identified a role of glucocorticoids in directly stimulating platelet production in vitro and in vivo (*61*). In line with our data, it was previously stated that glucocorticoids can induce cell cycle arrest in G_1_ in a variety of cancer cell lines (*75*, *76*), thus suggesting that increased platelet generation with dexamethasone treatment or serum starvation is caused by similar effects on cell cycle dynamics. In the future, a variety of novel tools such as FUCCI-based reporter mice for different cell cycle stages (*77*) in combination with intravital imaging will make it possible to further delineate cell cycle dynamics in MKs in vivo.

The findings presented in this manuscript not only are relevant for enhancing the efficacy of in vitro platelet production but also may open avenues for therapeutic options to treat thrombocytopenic patients with reduced proplatelet formation capacity despite normal or high MK counts as observed in ITP patients undergoing treatment with TPO mimetics such as eltrombopag or romiplostim (*78*). Consistent with this notion, previous studies have found that aged mice kept on a fasting-mimicking diet exhibited markedly increased platelet counts for the entirety of the fasting period (*79*), thus suggesting long-term benefits of caloric restriction on platelet production. Future research will address whether long-term fasting, which was previously also shown to protect rodents from cancer and heart disease, prolong lifespan (*80*), and increase stem cell regeneration and fitness (*81*), might be a potential adjuvant approach to increase platelet counts under disease settings.

Our findings are also of enhanced relevance in the context of a recently published meta-genome-wide association study, which identified 577 variants with implications on platelet counts (*82*). Among these variants, the study identified molecules involved in centrosome regulation (*CEP120*, *POC5*, *SPATC1*, and the kinesins *KIF1B* and *KIF26A*), the cell cycle (*CDK2AP1*, *CDKN1B*, *CABLES1*, *CDC7*, *PRC1*, and *MPHOSPH9*), and regulators of microtubule dynamics (*MAP1A* and *MAP1S*), which were all associated with altered platelet traits. While a plethora of these variants caused altered platelet counts due to systemic disease, 85 of the variants were identified independently and directly correlated to proteins expressed in MKs according to the Online Mendelian Inheritance in Man database. Of these, *CEP120*, *MPHOSPH9*, *CDK2AP1*, and *MAP1A* can be classified as potential regulators of platelet counts in vivo, possibly contributing to altered platelet production in humans.

In summary, we have provided insight into the mechanisms underlying the initiation of proplatelet formation and identified a previously undescribed cell cycle dependency of (pro)platelet production. Whether centrosomal clustering is causative for proplatelet formation or rather one of several intracellular cues warrants further investigation. Nevertheless, our findings will not only have implications for understanding what triggers platelet production but also have the potential to affect clinical efforts to increase platelet counts in thrombocytopenic patients.

## METHODS

### Statistics

Results are displayed as means ± SD from at least three independent biological replicates per group or as indicated. The distribution of data was assessed with a Shapiro-Wilk test. Differences were statistically analyzed using unpaired, two-tailed Student’s *t* test and one-or two-way analysis of variance (ANOVA) with Sidak correction for multiple comparisons. *P* < 0.05 was considered statistically significant: *P < 0.05; **P < 0.01; ***P < 0.001; ****P < 0.0001.

### Study approval

All animal work was approved by the International Animal Care and Use Committee at Boston Children’s Hospital, Boston, MA (protocol: 00002148). All human studies were approved by the Institutional Review Board at Boston Children’s Hospital (protocol: P00039193). Umbilical cord blood units were obtained from Pasquarello Tissue Bank (Dana Farber Cancer Institute) under informed consent.

### Mice

CD-1 [#CD1(ICR)] and C57BL/6N (#027C57BL/6) mice were acquired from Charles River Laboratories (Worcester, United States). *Kifc1*^−/−^ mice on a C57BL/6J background were generated in the Knockout Mouse Project by Baylor College and obtained from the University of Missouri Mutant Mouse Resource & Research Center (MMRRC #50739, United States). Both WT and *Kifc1*^−/−^ mice were provided by MMRRC. WT and *Kifc1*^−/−^ colonies were generated by crossing heterozygous *Kifc*^+/−^ mice to produce WT and *Kifc1*^−/−^ mice. Mice were housed in the animal facilities at Boston Children’s Hospital, Boston, MA.

### Short-term starvation

For STS experiments, mice were housed in cages without food but with water ad libitum for 48 hours. Body weight and behavior were assessed every 12 hours, and mice were euthanized upon severe lethargy or loss of more than 20% of body weight. Blood was collected at 0, 12, 24, 36, and 48 hours via laceration of the lateral tail veins into EDTA-coated tubes, and blood cell parameters were assessed using an automated blood cell analyzer (Sysmex Corporation). Mice were euthanized at 48 hours by CO_2_ asphyxiation.

### Genotyping

Deletion of *Kifc1* was assessed by PCR in DNA retrieved from ear punches, which were lysed using DirectPCR lysis reagent (102-T, Viagen Biotech). Samples were directly used for PCR using the following primers and a KOD DNA polymerase (71085, Sigma-Aldrich): F2, 5′-CACATCCGGGGACTTTCACA-3′; Mut R2, 5′-GGACAGATGTGCCCTAGCTT-3′; WT R1, 5′-CCAGCGTTTGAGTCTTCTCC-3′.

### Blood parameters

Mice were anesthetized using isoflurane and retro-orbitally bled into EDTA-coated tubes. For consecutive measurements, blood was retrieved via laceration of the lateral tail veins into EDTA-coated tubes. Blood parameters were assessed using an automated blood cell analyzer (Sysmex Corporation).

### Platelet depletion

Mice were bled retro-orbitally into EDTA-coated tubes using heparinized capillaries, blood was diluted in isotonic saline [0.9% (w/v) of sodium chloride], and platelet counts were assessed using an automated blood cell analyzer (Sysmex Corporation). Subsequently, mice were retro-orbitally injected with platelet depletion antibody (2 μg g^−1^; #R300, Emfret Analytics) or a respective antibody control (C301, Emfret Analytics), and platelet depletion was verified on day 1. Platelet counts were measured on consecutive days afterward. Mice were euthanized by CO_2_ asphyxiation upon observation of platelet recovery (96 hours).

### Platelet lifespan

For platelet lifespan analysis, mice were retro-orbitally injected with Biotin-X-NHS (203189, EMD Millipore) at 4 mg ml^−1^ twice in the span of 1 hour to achieve a concentration of 10 mg ml^−1^ in circulation. The amount of biotin-labeled platelets was assessed by flow cytometry (Accuri C6 plus, BD Biosciences) using Alexa Fluor 488–labeled streptavidin (S11223, Sigma-Aldrich).

### Platelet activation and TO staining

Mice were bled up to 70 μl into EDTA-coated tubes under isoflurane anesthesia. Blood was washed twice in Hepes-buffered Tyrode’s solution (J67607, Alfa Aesar) without Ca^2+^, followed by centrifugation at 750*g* for 5 min. After the last washing step, 50 μl of washed blood was resuspended in Hepes-buffered Tyrode’s containing 2 mM Ca^2+^. Fifty microliters of washed blood was added to fluorophore-conjugated antibodies against activated α_IIb_β_3_ integrins (JON/A-PE, Emfret Analytics) and P-selectin [WUG 1.9–fluorescein isothiocyanate (FITC), Emfret Analytics]. Subsequently, platelets were stimulated with different agonists (adenosine diphosphate, U46619, thrombin, CRP-XL). Unstimulated platelets served as a resting control. Platelets were incubated for 15 min at room temperature in the dark. The reaction was stopped by addition of 500 μl of phosphate-buffered saline (PBS) to each tube. MFI was assessed by flow cytometry (Accuri C6 plus, BD Biosciences). For analysis of TO-positive platelets, 5 μl of unwashed whole blood was diluted in 500 μl of Hepes-buffered Tyrode’s. Fifty microliters of diluted blood was incubated with TO (200 ng ml^−1^) for 15 min at room temperature in the dark and diluted with 500 μl of PBS. The percentage of TO^high^ platelets was determined by flow cytometry (Accuri C6 plus, BD Biosciences).

### Culture of murine FLMKs and BM-derived MKs

Pregnant CD-1 mice were euthanized by CO_2_ asphyxiation and fetal liver cells were isolated and cultured in Dulbecco’s modified Eagle’s medium (DMEM) containing TPO (50 ng ml^−1^) for 72 or 96 hours as indicated (*83*). For isolation of BM cells, C57BL/6J mice were euthanized by CO_2_ asphyxiation, and humeri, femora, tibiae, and iliac crests were removed. Cells were isolated by centrifugation for 40 s at 2500*g* (*84*). Cells were labeled using a biotin rat anti-mouse lineage panel (133307, BioLegend) and magnetic anti-rat Dynabeads (11415D, Thermo Fisher Scientific). Cells were cultured in DMEM containing TPO (50 ng ml^−1^) and 100 anti-thrombin units of recombinant hirudin (ARE120A, Aniara Diagnostic) for 72 hours. Mature MKs were retrieved using density gradient enrichment as previously described (*83*).

### Culture of human umbilical cord blood–derived MKs

Cord blood was diluted, and white blood cells were isolated by Ficoll gradient centrifugation. Hematopoietic stem and progenitor cells were enriched using a CD34 Microbead kit (130-046-702, Miltenyi Biotec) and cultured in StemSpan SFEM II medium (09605, STEMCELL Technologies) containing stem cell factor (100 ng ml^−1^; 300-07, PeproTech), human thrombopoietin (hTPO) (100 ng ml^−1^; 300-18, PeproTech), Flt-3 (100 ng ml^−1^; 300-19, PeproTech), and interleukin-6 (100 ng ml^−1^; 200-06, PeproTech) for 6 days. On day 6, expansion medium was switched to differentiation medium containing only hTPO (50 ng ml^−1^). Cells were stained using SiR-tubulin (CY-SC002, Cytoskeleton) on day 12 and imaged by live-cell spinning-disk confocal microscopy to visualize clustering.

### Retroviral expression of fluorescent proteins in FLMKs

EB3-GFP construct was generated as previously described (*7*, *85*). Centrin2 C-terminally fused to GFP in pEGFP-N1 plasmid was a gift from Kengaku and colleagues (*86*). Centrin2-GFP was PCR-amplified from plasmid using following primers containing Eco R1 sites: forward, 5′-AAGAATTCGCCGCCATGGCCTCTAATTTTAAGAA-3′; reverse, 5′-TTGAATTCTTACTTGTACAGCTCGTcCATGCCGA-3′.

PCR product and MSCV puro backbone (Addgene #68469) were digested with Eco R1 and ligated to generated MSCV-Centrin2-GFP. pENTR-PIP-FUCCI (Addgene #118621) was recombined with MSCV-N-FLAG-HA-IRES-PURO (Addgene #41033) to generate MSCV-FUCCI using LR clonase (11791020, Invitrogen). MSCV expressing β1-tubulin fused to dendra2 (MSCV–β1-tubulin–dendra2) was generated as described previously (*10*). Fetal liver–or BM-derived progenitor cells were transduced and cultured in medium containing TPO (50 ng ml^−1^) for 4 days. MKs were enriched by density gradient sedimentation, stained with Hoechst (5 μg ml^−1^; 33342, Invitrogen), and microtubule structures were imaged using live-cell spinning-disk confocal microscopy before proplatelet formation or during proplatelet formation as indicated (Yokogawa spinning disk confocal on an inverted Nikon Ti fluorescence microscope with incubation enclosure; 20× objective). Clustered centrosome status was quantified manually using Atom and ImageJ software (version 2.1.0/1.53c). FLMKs transduced with MSCV-PIP-FUCCI were stained with SiR-tubulin (CY-SC002, Cytoskeleton) to visualize microtubules after density gradient enrichment and imaged by live-cell spinning-disk confocal microscopy to visualize active clustering. The number of MKs containing clustered centrosomes was manually quantified for each cell cycle stage using ImageJ software (version 2.1.0/1.53c).

### Live-cell imaging of proplatelet formation

FLMKs or BM MKs were isolated as described. Immediately following density gradient enrichment, MKs were treated with CCB02 (2100864-57-9, MedChemExpress), SR (AOB0951, Aobious), AZ82 (533916, Sigma-Aldrich), CW069 (S7336, Selleckchem), or Gris (S4071, Selleckchem) at the indicated concentrations or cultured in serum-free medium and proplatelet formation was visualized on an IncuCyte imaging system and quantified using a custom image analysis pipeline (*87*).

### Cytotoxicity assay

The lactate dehydrogenase cytotoxicity assay was performed according to the manufacturer’s instructions (C20300, Thermo Fisher Scientific). Briefly, progenitor cells were cultured for 3 days, or BM MKs were cultured overnight in the presence of DMSO, SR (AOB0951, Aobious), CW069 (S7336, Selleckchem), and CCB02 (2100864-57-9, MedChemExpress). Lactate dehydrogenase activity in the supernatant was assayed the following day. Serum-free DMEM, DMEM containing 10% fetal bovine serum, and untreated cells served as controls.

### Ploidy

MK polyploidization was assessed in BM MKs in vitro and ex vivo. MKs were treated with DMSO, SR (AOB0951, Aobious), or Gris (S24071, Selleckchem) on day 0, and cells were retrieved on day 4. Cells were washed in PBS and fixed and permeabilized in 100% (v/v) of ethanol for 30 min on ice. Cells were then treated with ribonuclease A (EN0531, Thermo Fisher Scientific) and stained with an anti-CD41-APC antibody (133914, BioLegend) and propidium iodide (PI) (P1304-MP, Sigma-Aldrich) for 30 min on ice. Ploidy distribution and percentage of CD41-positive cells were analyzed using a spectral flow cytometer (Cytek Biosciences). For native BM MKs, BM was isolated from one femur by centrifugation at 2500*g* for 40 s. BM was filtered through a 100-μm cell strainer, and red blood cells were lysed using ACK buffer (A1049201, Gibco). BM cells were fixed in ethanol and stained as described above.

### Flow cytometry on mature BM-derived MKs

BM MKs were isolated and cultured as described above for 4 days. Cells were pelleted and stained for CD41 (133904, BioLegend) and CD42d (148504, BioLegend) on ice for 30 min. The number of single- and double-positive cells and the MFI of CD42d/GPV were analyzed by flow cytometry (Accuri C6 plus, BD Biosciences).

### Immunofluorescence staining of MKs

FLMKs were isolated as described above. Four hours after density gradient enrichment, MKs were fixed and spun down onto coverslips, stained with antibodies against EB1 (610534, BD Biosciences) and pericentrin (ab4448, Abcam), and imaged using a spinning-disk confocal microscope (Yokogawa spinning disk confocal on an inverted Nikon Ti fluorescence microscope with incubation enclosure; 20× objective). BM MKs were incubated in the presence of TPO and recombinant hirudin as described above. Density gradient separation was performed on early day 4, and, for proplatelet formation assays, enriched MKs were incubated for 24 hours on CD31-coated (102502, BioLegend) eight-well chambered coverslips (155409, Thermo Fisher Scientific). MKs were fixed using 4% (w/v) of paraformaldehyde (PFA) in PBS containing 0.1% (v/v) of Tween 20, and nonspecific antibody binding was blocked using 3% (w/v) of bovine serum albumin in PBS. To analyze cells in solution, MKs were resuspended in medium after density gradient enrichment and fixed in 4% (w/v) of PFA in PBS containing 0.1% (v/v) of Tween 20 2 hours later. MKs on coverslips and in solution were stained for pericentrin (ab4448, Abcam), F-actin (Phalloidin Atto647N, 65906, Sigma-Aldrich), CD42a/GPIX (M-051-0, Emfret Analytics), GM130 (NBP2-53420, Novus Biologicals), cyclin D1 (ab134175, Abcam), Ki67 (13-5698-82, Invitrogen), or α-tubulin–Alexa Fluor 488 (MA-1-38000-A488, Invitrogen). Nuclei were counterstained using 4′,6-diamidino-2-phenylindole (DAPI). Images were acquired at a Zeiss LSM880 (40× objective). Proplatelet tip number and size were quantified using the cell counter plugin in ImageJ (version 2.1.0/1.53c).

### Cryosections and immunostainings

Femora were isolated and fixed in 4% (w/v) of PFA in PBS overnight, after which they were transferred into 10% (w/v) of sucrose in PBS. After two additional steps of increasing sucrose concentrations (10 to 30%), femora were embedded in a water-soluble embedding medium and frozen down at −20°C. A tape transfer system (*88*) was used to retrieve 10-μm sections using a cryostat (Leica Biosystems). Sections were rehydrated in PBS for 20 min, and nonspecific antibody binding was blocked using 5% (v/v) of donkey serum. Sections were stained for pericentrin (ab4448, Abcam), CD105 (AF1320, R&D Systems), CD41 (133902, BioLegend), laminin (L9393, Sigma-Aldrich), CD42a/GPIX (M-051-0, Emfret Analytics), or α-tubulin–Alexa Fluor 488 (MA-1-38000-A488, Invitrogen). Nuclei were counterstained using DAPI. Slides were washed and mounted using Fluoroshield mounting medium (F6182, Sigma-Aldrich). Representative images were acquired at a Zeiss LSM880 confocal microscope (40× objective). Whole sections were imaged using an automated image platform (Lionheart, BioTek; 4× objective). Supercentrosomes were quantified manually using ImageJ software (version 2.1.0/1.53c).

### Proteome profiler cytokine array and enzyme-linked immunosorbent assays

BM supernatant was retrieved by centrifugation (2500*g* for 40 s) of femora and tibiae into 100 μl of PBS as previously described (*84*). Supernatant was immediately frozen and spun at 10,000 rpm for 1 min before usage. Plasma was isolated from EDTA-diluted whole blood by consecutive centrifugations at 750*g* and 10,000*g*. BM and blood cytokines were analyzed in pooled samples using a Proteome Profiler Cytokine Array (ARY006, R&D Systems). TPO and cortisol levels in blood plasma were determined using the Quantikine ELISA Kit (MTP00, R&D Systems) and the Cortisol Parameter Assay Kit (KGE008B, R&D Systems), respectively.

### Ubiquitin pull-down

Poly-ubiquitinated proteins were enriched and isolated using the Ubiquitin Enrichment Kit (89899, Thermo Fisher Scientific) according to the manufacturer’s instructions. Briefly, lysates of untreated MKs or MKs treated with the proteasome inhibitor MG132 containing 15 μg of protein were transferred onto spin columns and rotated overnight at 4°C. Samples are eluted after several washing steps and transferred onto polyvinylidene difluoride membranes. SDS–polyacrylamide gel electrophoresis was conducted as described above.

## Supplementary Materials

### This PDF file includes:

Figs. S1 to S4 Table S1 Legends for movies S1 to S10 References
