# Supplementary material for: Cell cycle–dependent centrosome clustering precedes proplatelet formation
Source: Sci Adv. 2024 Jun 19;10(25):eadl6153. doi: 10.1126/sciadv.adl6153 (PMC11186502; doi:10.1126/sciadv.adl6153)
Supplement: Supplementary file 1 — Figs. S1 to S4 Table S1 Legends for movies S1 to S10 References [file sciadv.adl6153_sm.pdf]

Science Advances
AAAS

# Supplementary Materials for

## Cell cycle–dependent centrosome clustering precedes proplatelet formation

Isabelle C. Becker *et al.*

Corresponding author: Joseph E. Italiano, joseph.italiano@childrens.harvard.edu

*Sci. Adv.* **10**, eadl6153 (2024)
DOI: 10.1126/sciadv.adl6153

**The PDF file includes:**

Figs. S1 to S4
Table S1
Legends for movies S1 to S10
References

**Other Supplementary Material for this manuscript includes the following:**

Movies S1 to S10

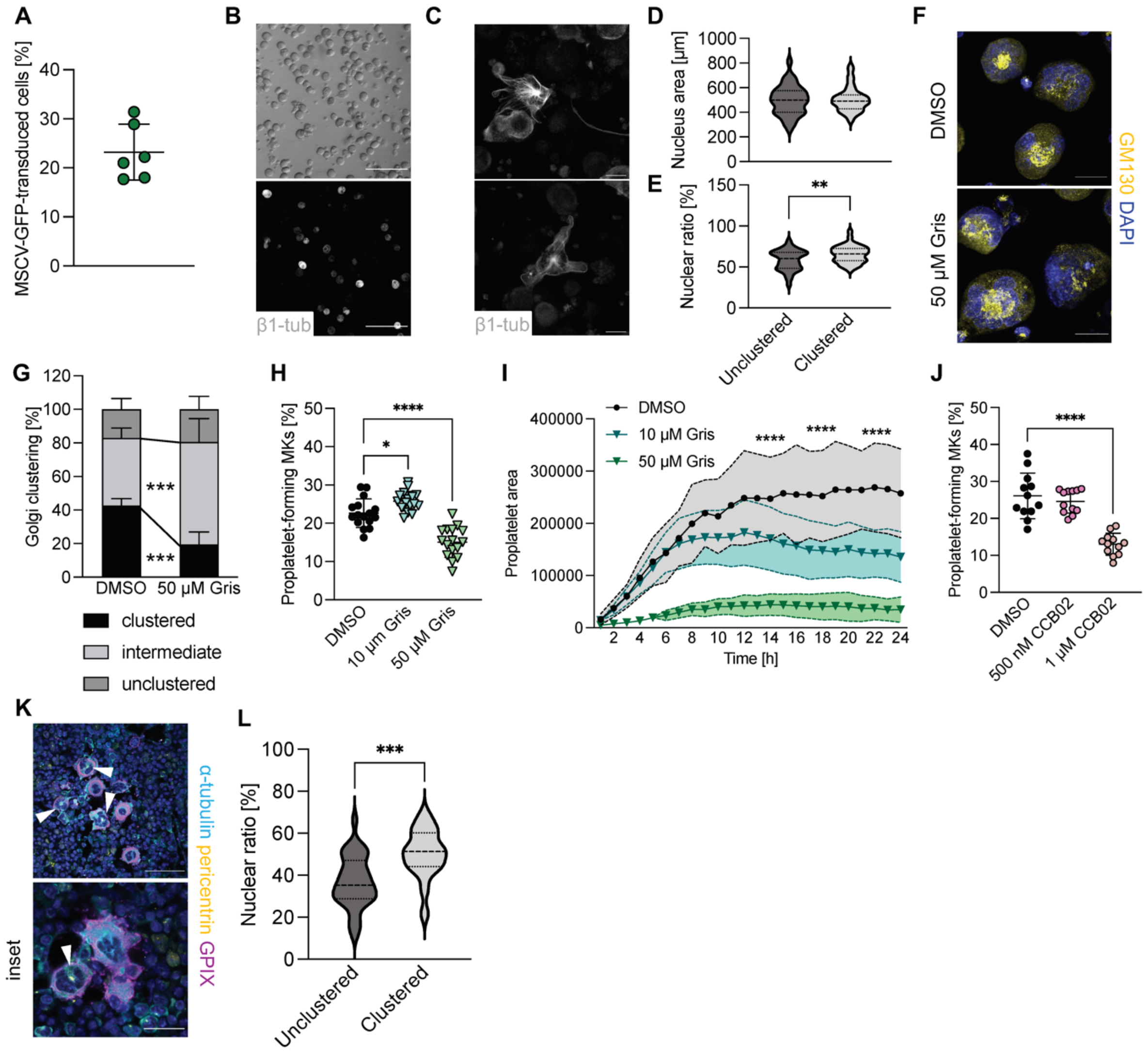


**Fig S1.** (**A)** Flow cytometric analysis of cells transduced with an empty MSCV-EGFP vector control. N=6. **(B)** Representative images of transduction efficiency of FLMKs transduced with MSCV-β1-tubulin-dendra2. Scale bars: 150 µm. **(C)** Representative images of MSCV-β1-tubulin-dendra2-transduced FLMKs containing clustered centrosomes during initiation of proplatelet formation. Scale bars: 20 µm. **(D, E)** Nucleus area **(D)** and relation to cell size **(E)** was analyzed in MKs with unclustered vs. clustered centrosomes. N=2. At least 50 cells were analyzed per mouse. Unpaired, two-tailed student's t-test. P < 0.01 **. **(F, G)** Visualization and quantification of Golgi ribbon formation in BMMKs treated with DMSO or 50 µM Gris for 6 hours. Golgi apparatus was visualized using an antibody against the Golgi membrane protein GM130. Golgi distribution was quantified manually using ImageJ Software. Scale bars: 20 µm. N=3. At least 50 cells were analyzed per mouse. Two-way ANOVA with Sidak correction for multiple comparisons. P < 0.001 ***. **(H, I)** Percentage of proplatelet-forming MKs at 24h and proplatelet area upon treatment with DMSO, 10 or 50 µM Gris was assessed using an automated imaging platform and custom analysis pipeline [87]. N=2; 4 technical replicates. One-way ANOVA with Sidak correction for multiple comparisons. P < 0.0001 ****. **(J)** Percentage of proplatelet-forming MKs at 24h upon treatment with

CCB01. One-way ANOVA with Sidak correction for multiple comparisons. $P < 0.0001$ ****. **(K)** Representative images of MKs containing clustered centrosomes in situ. Arrows point at clustered centrosomes. Scale bars: 50 µm. **(L)** DNA area in relation to cell size was analyzed in MKs with unclustered vs. clustered centrosomes in situ. N=4. At least 20 cells were analyzed per mouse. Unpaired, two-tailed student's t-test. $P < 0.001$ ***. All data are presented as mean ± SD.

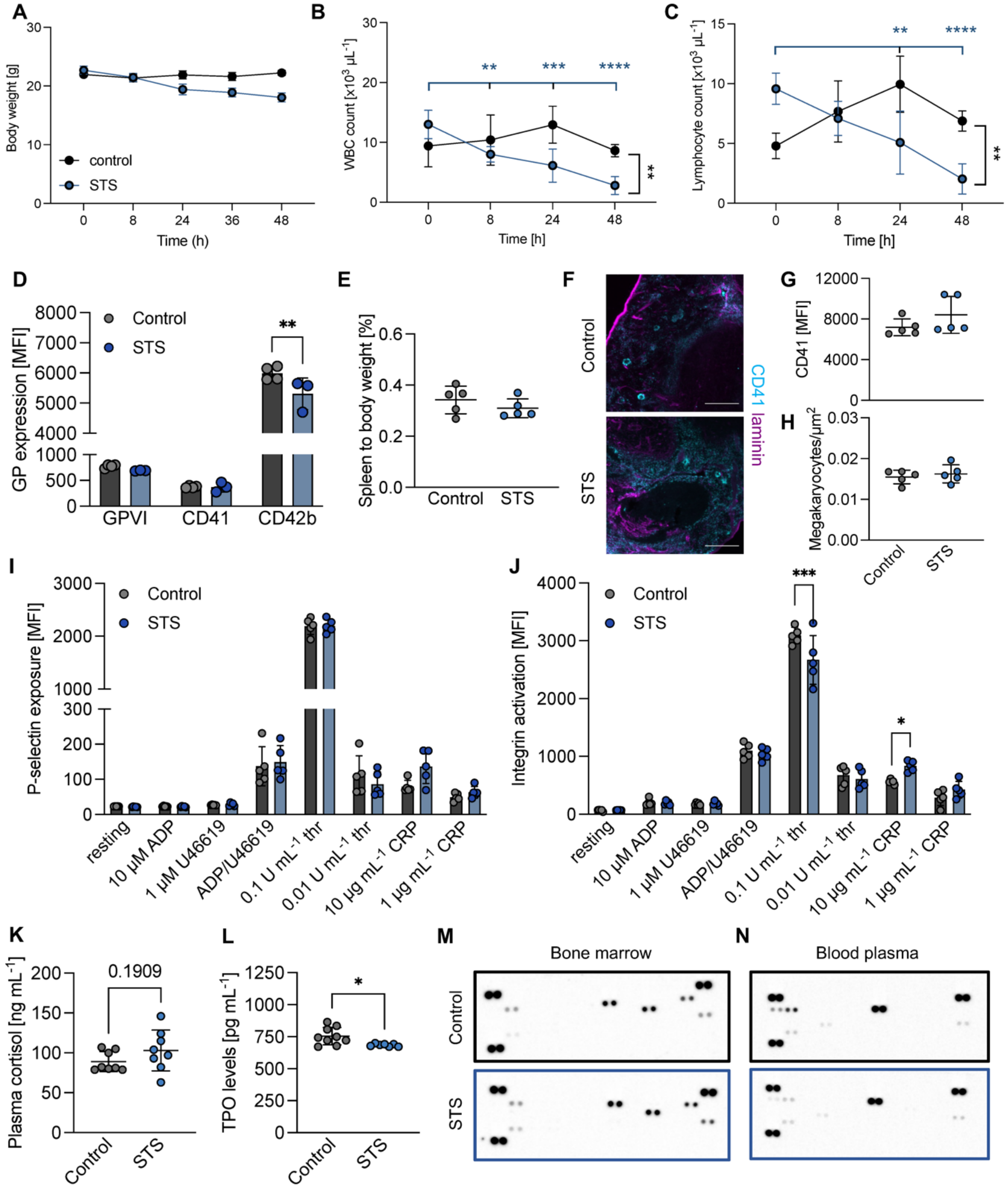


**Fig S2.** **(A)** Body weight of control and STS mice was measured at the indicated timepoints. N=5. Data are presented as mean ± SD. **(B, C)** White blood cell and lymphocyte counts of control and STS mice were assessed using an automated blood cell analyzer. N=5. One-way ANOVA with Sidak correction for multiple comparisons. $P < 0.0001$ ****. **(D)** Glycoprotein (GP) expression of control and STS platelets was assessed by flow cytometry. N=4. Two-way ANOVA with Sidak correction for multiple comparisons. $P < 0.01$ **. **(E)** Spleen weight was normalized to body weight. N=5. Data are presented as mean ± SD. **(F)** Spleens from control and STS mice were sectioned, stained for laminin and CD41 and imaged using an

automated imager. Scale bars: 100 μm. **(G, H)** CD41 MFI and MK numbers were assessed using an automated image analysis software. N=5. **(I, J)** Platelet integrin activation and P-selectin exposure were analyzed by flow cytometry upon stimulation with different agonists. N=5. Multiple student's t-tests. $P < 0.05$ *; $P < 0.001$ ***. **(K, L)** Plasma cortisol and TPO levels in control and STS mice was assessed using enzyme-based immunosorbent assays. N=9. Unpaired, two-tailed student's t-test. $P < 0.05$ *. **(M, N)** Bone marrow and blood plasma were isolated from control and STS mice and subjected to cytokine profiling. Plasma was pooled from 9 mice. All data are presented as mean ± SD.

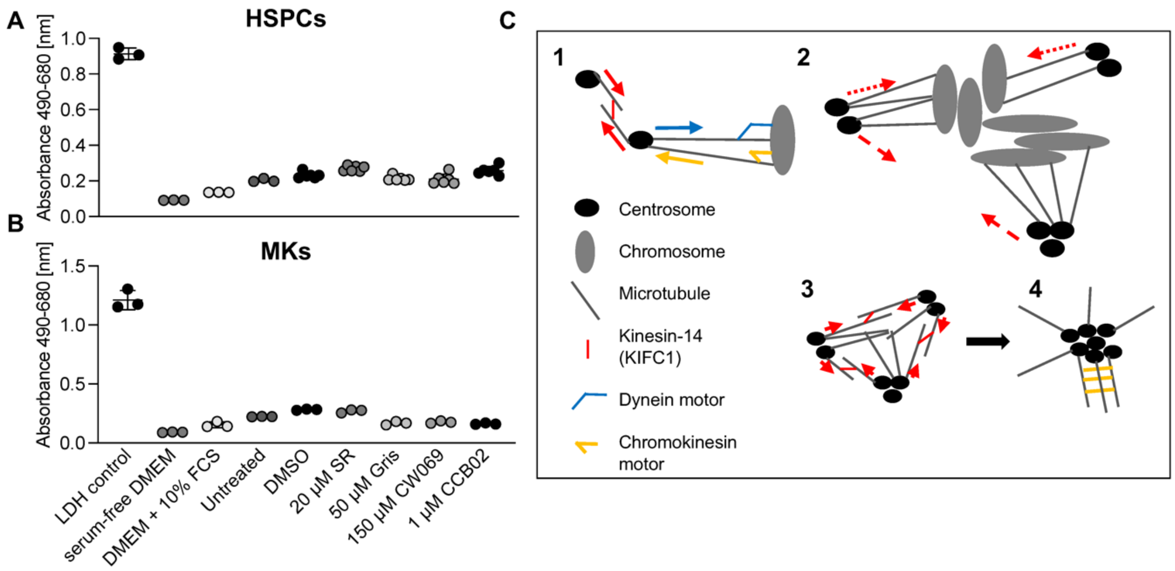


**Fig S3. (A, B)** Cytotoxicity of the inhibitors SR31527 (SR), CW069, griseofulvin (Gris) and CCB02 in hematopoietic stem and progenitor cells (HSPCs) cultured for 3 days **(A)** as well as mature BMMKs cultured for 24h **(B)** was assessed using an LDH cytotoxicity assay per the manufacturers' instructions. Absorbance was measured at a plate reader. **(A)** N=2; **(B)** N=1; 3 technical replicates. Data are presented as mean ± SD. **(C)** Simulation-based modeling of interactions between chromosomes and centrosomes [64, 65]: Kinesin-14 motors like KIFC1 (red) stretching from two centrosomes generate effective inter-centrosomal attraction forces (red arrows) (1). Microtubules interacting with dynein motors (blue) on the kinetochores are pulled toward the chromosomes causing the effective attraction force between the centrosome and chromosome (blue arrow). Microtubules interacting with chromokinesin motors (purple) on the chromosome arms are pushed away from the chromosomes causing the effective repulsion force between the centrosome and chromosome (purple arrow). Two opposing centrosome-chromosome forces position centrosomes at a characteristic distance from the chromosomes (2). The chromosomes aggregate to several clusters at the center, while the effective attraction clusters some of the centrosomes and localizes them around the chromosomes resulting in multipolarity. The centrosomal clusters do not aggregate further because the attraction between them is either blocked by the chromosomes in between (dotted arrows) or is too weak because of the large inter-centrosomal cluster distances (dashed arrows). Following DNA decondensation, the only remaining force is the attraction between the centrosomal clusters (3). This KIFC1-generated attraction force clusters all centrosomes together. The resulting combined microtubule aster is so dense that crosslinking action of MAPs could lead to bundling of microtubules (4), from which they are distributed to the cell cortex.

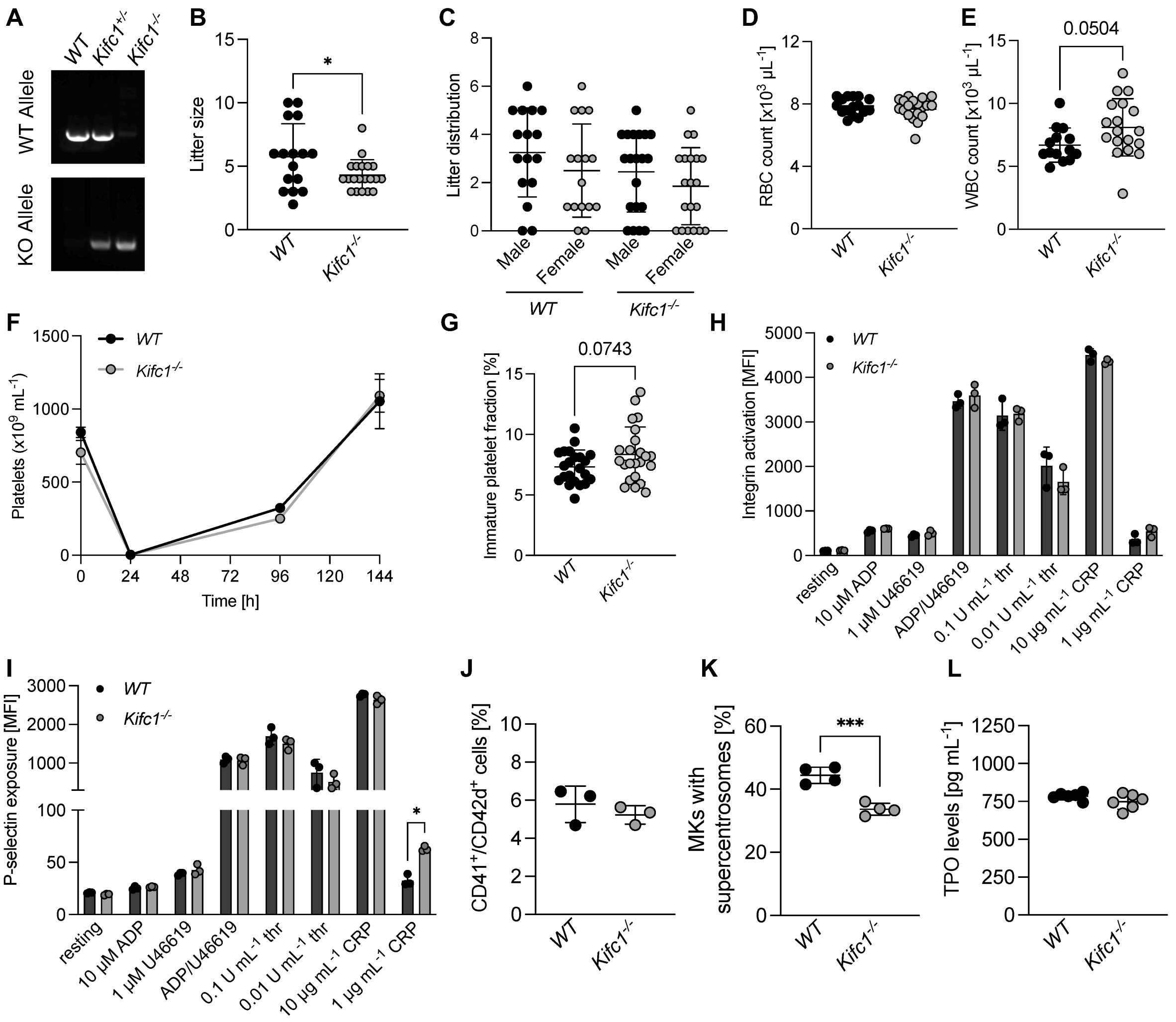


**Fig S4. (A)** PCR for wildtype (WT) and mutant (KO) *Kifc1* allele using DNA derived from *Kifc1*$^{-/-}$, heterozygous (*Kifc*$^{+/-}$) and *WT* mice. **(B, C)** Analysis of litter sizes **(B)** and sex distribution among the litters **(C)** of *WT* and *Kifc1*$^{-/-}$ mice. Unpaired, two-tailed student's t-test. P < 0.05. **(D, E)** Red and white blood cell counts in *WT* and *Kifc1*$^{-/-}$ mice were assessed using an automated blood cell analyzer. N=25. Unpaired, two-tailed student's t-test. **(F)** Platelet recovery after depletion was assessed using an automated blood cell analyzer. N=3. **(G)** Immature platelet fraction in *WT* and *Kifc1*$^{-/-}$ mice was assessed using an automated blood cell analyzer. N=25. Unpaired, two-tailed student's t-test. **(H, I)** Activation of platelet $\alpha_{IIb}\beta_3$ integrins and exposure of P-selectin were analyzed by flow cytometry upon stimulation with different agonists. N=4. Unpaired, two-tailed student's t-test. P < 0.05. **(J)** Percentage of MKs expressing CD41 and CD42d after 4 days of culturing was assessed by flow cytometry. N=3. **K)** Number of MKs containing clustered centrosomes was quantified by immunofluorescence stainings for pericentrin and α-tubulin. N=4. Unpaired, two-tailed student's t-test. P < 0.001 ***. **(L)** TPO levels in plasma derived from WT or *Kifc1*$^{-/-}$ mice were analyzed using an enzyme-linked immunosorbent assay. N=6. All data are presented as mean ± SD.

**Table S1.**

| Gene | Protein | Method | Function |
|---|---|---|---|
| **Tpx2** | Microtubule Nucleation Factor | Ubiquitin Pulldown & Polysome Profiling [48] | Spindle assembly factor required for normal assembly of mitotic spindles. Mediates AURKA localization to spindle microtubules [89]. |
| **Aurka** | Aurora Kinase A | Polysome Profiling [48] | Associates with the centrosome and the spindle microtubules during mitosis and plays a critical role in various mitotic events [90]. |
| **Aurkb** | Aurora Kinase B | Polysome Profiling [48] | Kinase component of the chromosomal passenger complex (CPC), a key regulator of mitosis [90]. |
| **Plk1, 4** | Polo-like Kinase 1, 4 | Polysome Profiling [48] | Regulates centrosome maturation, spindle assembly, and cytokinesis. Contributes to the regulation of AURKA function [91]. |
| **Kif11** | Kinesin Family Member 11 | Polysome Profiling [48] | Motor protein required for establishing a bipolar spindle during mitosis [92]. |
| **Kif2a** | Kinesin Family Member 2a | Ubiquitin Pulldown | Plus-end-directed microtubule-dependent motor required for progression through mitosis [93]. |
| **StarD9** | STAR Related Lipid Transfer Domain Containing 9 | Ubiquitin Pulldown | Microtubule-dependent motor protein required for spindle pole assembly during mitosis and to stabilize the pericentriolar material (PCM) [94]. |
| **CEP192** | Centrosomal Protein 192 | Ubiquitin Pulldown | Required for mitotic centrosome and spindle assembly. Appears to be a major regulator of PCM recruitment and centrosome maturation [95]. |

**Table S1.** Cell cycle proteins identified by polysome profiling [48] and ubiquitin pulldown.

**Movie S1.** Timelapse of MSCV-Centrin2-GFP-transduced FLMKs imaged every 10 min for 2 hours at a Yokogawa spinning disk confocal/inverted Nikon Ti fluorescence microscope with incubation enclosure (20x objective). Scale bar: 5 µm.

**Movie S2.** Timelapse of MSCV-FUCCI-transduced FLMKs imaged every hour for 24 hours at a Yokogawa spinning disk confocal/inverted Nikon Ti fluorescence microscope with incubation enclosure (20x objective). Scale bar: 30 µm.

**Movie S3.** Timelapse of MSCV-FUCCI-transduced FLMKs imaged every hour for 24 hours at a Yokogawa spinning disk confocal/inverted Nikon Ti fluorescence microscope with incubation enclosure (20x objective). Video shows MKs transitioning to proplatelet formation. Scale bar: 60 µm.

**Movie S4.** Timelapse of MSCV-β1-tubulin-dendra2-transduced FLMKs imaged every hour for 24 hours at a Yokogawa spinning disk confocal/inverted Nikon Ti fluorescence microscope with incubation enclosure (20x objective). Video shows MKs prior to proplatelet formation. Scale bar: 50 µm.

**Movie S5.** Timelapse of MSCV-β1-tubulin-dendra2-transduced FLMKs imaged every hour for 24 hours at a Yokogawa spinning disk confocal/inverted Nikon Ti fluorescence microscope with incubation enclosure (20x objective). Video shows MKs during proplatelet formation. Scale bar: 50 µm.

**Movie S6, 7.** Timelapse of MSCV-β1-tubulin-dendra2-transduced FLMKs imaged every hour for 24 hours at a Yokogawa spinning disk confocal/inverted Nikon Ti fluorescence microscope with incubation enclosure (20x objective). Video shows MKs transitioning to proplatelet formation. Scale bar: 5 µm.

**Movie S8.** Timelapse of FLMKs transduced with SFV-EB3-GFP visualizing EB3 comets emanating from clustered centrosomes towards the cell cortex [7]. MKs were imaged every 5 sec at a Zeiss Axiovert 200 microscope (63x objective).

**Movie S9.** Proplatelet formation of DMSO-treated FLMKs was imaged using the Incucyte imaging system over 24 hours. Scale bar: 150 µm.

**Movie S10.** Proplatelet formation of FLMKs treated with 40 µm SR31527 was imaged using the Incucyte imaging system over 24 hours. Scale bar: 150 µm.

## REFERENCES AND NOTES


1. N. L. Asquith, E. Carminita, V. Camacho, A. Rodriguez-Romera, D. Stegner, D. Freire, I. C. Becker, K. R. Machlus, A. O. Khan, J. E. Italiano, The bone marrow is the primary site of thrombopoiesis. *Blood* **143**, 272–278 (2024).

2. K. R. Machlus, J. E. Italiano, Jr., The incredible journey: From megakaryocyte development to platelet formation. *J. Cell Biol.* **201**, 785–796 (2013).

3. K. R. Machlus, J. N. Thon, J. E. Italiano, Jr., Interpreting the developmental dance of the megakaryocyte: A review of the cellular and molecular processes mediating platelet formation. *Br. J. Haematol.* **165**, 227–236 (2014).

4. R. P. Becker, P. P. De Bruyn, The transmural passage of blood cells into myeloid sinusoids and the entry of platelets into the sinusoidal circulation; a scanning electron microscopic investigation. *Am. J. Anat.* **145**, 183–205 (1976).

5. C. Bennett, M. Lawrence, J. A. Guerrero, S. Stritt, A. K. Waller, Y. Yan, R. W. Mifsud, J. Ballester-Beltrán, A. Baig, A. Mueller, L. Mayer, J. Warland, C. J. Penkett, P. Akbari, T. Moreau, A. L. Evans, S. Mookerjee, G. J. Hoffman, K. Saeb-Parsy, D. J. Adams, A. L. Couzens, M. Bender, W. N. Erber, B. Nieswandt, R. J. Read, C. Ghevaert, CRLF3 plays a key role in the final stage of platelet genesis and is a potential therapeutic target for thrombocythemia. *Blood* **139**, 2227–2239 (2022).

6. F. Tablin, M. Castro, R. M. Leven, Blood platelet formation in vitro. The role of the cytoskeleton in megakaryocyte fragmentation. *J. Cell Sci.* **97**, 59–70 (1990).

7. S. R. Patel, J. L. Richardson, H. Schulze, E. Kahle, N. Galjart, K. Drabek, R. A. Shivdasani, J. H. Hartwig, J. E. Italiano, Jr., Differential roles of microtubule assembly and sliding in proplatelet formation by megakaryocytes. *Blood* **106**, 4076–4085 (2005).

8. J. N. Thon, H. Macleod, A. J. Begonja, J. Zhu, K. C. Lee, A. Mogilner, J. H. Hartwig, J. E. Italiano Jr, Microtubule and cortical forces determine platelet size during vascular platelet production. *Nat. Commun.* **3**, 852 (2012).

9. A. O. Khan, A. Slater, A. Maclachlan, P. L. R. Nicolson, J. A. Pike, J. S. Reyat, J. Yule, R. Stapley, J. Rayes, S. G. Thomas, N. V. Morgan, Post-translational polymodification of β1-tubulin regulates motor protein localisation in platelet production and function. *Haematologica* **107**, 243–259 (2022).

10. M. Bender, J. N. Thon, A. J. Ehrlicher, S. Wu, L. Mazutis, E. Deschmann, M. Sola-Visner, J. E. Italiano, J. H. Hartwig, Microtubule sliding drives proplatelet elongation and is dependent on cytoplasmic dynein. *Blood* **125**, 860–8 (2015).

11. S. Mbiandjeu, A. Balduini, A. Malara, Megakaryocyte cytoskeletal proteins in platelet biogenesis and diseases. *Thromb. Haemost.* **122**, 666–678 (2022).

12. K. S. Potts, A. Farley, C. A. Dawson, J. Rimes, C. Biben, C. de Graaf, M. A. Potts, O. J. Stonehouse, A. Carmagnac, P. Gangatirkar, E. C. Josefsson, C. Anttila, D. Amann-Zalcenstein, S. Naik, W. S. Alexander, D. J. Hilton, E. D. Hawkins, S. Taoudi, Membrane budding is a major mechanism of in vivo platelet biogenesis. *J. Exp. Med.* **217**, e20191206 (2020).

13. E. Brown, L. M. Carlin, C. Nerlov, C. Lo Celso, A. W. Poole, Multiple membrane extrusion sites drive megakaryocyte migration into bone marrow blood vessels. *Life Sci. Alliance* **1**, e201800061 (2018).

14. S. Nishimura, M. Nagasaki, S. Kunishima, A. Sawaguchi, A. Sakata, H. Sakaguchi, T. Ohmori, I. Manabe, J. E. Italiano Jr, T. Ryu, N. Takayama, I. Komuro, T. Kadowaki, K. Eto, R. Nagai, IL-1α induces thrombopoiesis through megakaryocyte rupture in response to acute platelet needs. *J. Cell Biol.* **209**, 453–66 (2015).

15. K. Kaushansky, Historical review: Megakaryopoiesis and thrombopoiesis. *Blood* **111**, 981–986 (2008).

16. S. J. Mowla, M. R. P. Sapiano, J. M. Jones, J. J. Berger, S. V. Basavaraju, Supplemental findings of the 2019 national blood collection and utilization survey. *Transfusion* **61**(Suppl. 2), S11–S35 (2021).

17. K. Kaushansky, The molecular mechanisms that control thrombopoiesis. *J. Clin. Invest.* **115**, 3339–3347 (2005).

18. J. B. Bussel, G. Soff, A. Balduzzi, N. Cooper, T. Lawrence, J. W. Semple, A review of romiplostim mechanism of action and clinical applicability. *Drug Des. Devel. Ther.* **15**, 2243–2268 (2021).

19. W. B. Mitchell, J. B. Bussel, Thrombopoietin receptor agonists: A critical review. *Semin. Hematol.* **52**, 46–52 (2015).

20. W. Vainchenker, H. Raslova, Megakaryocyte polyploidization: Role in platelet production. *Platelets* **31**, 707–716 (2020).

21. H. K. Matthews, C. Bertoli, R. A. M. de Bruin, Cell cycle control in cancer. *Nat. Rev. Mol. Cell Biol.* **23**, 74–88 (2022).

22. K. Ravid, J. Lu, J. M. Zimmet, M. R. Jones, Roads to polyploidy: The megakaryocyte example. *J. Cell. Physiol.* **190**, 7–20 (2002).

23. Y. Zhang, Z. Wang, D. X. Liu, M. Pagano, K. Ravid, Ubiquitin-dependent degradation of cyclin B is accelerated in polyploid megakaryocytes. *J. Biol. Chem.* **273**, 1387–92 (1998).

24. Y. Zhang, Z. Wang, K. Ravid, The cell cycle in polyploid megakaryocytes is associated with reduced activity of cyclin B1-dependent Cdc2 kinase. *J. Biol. Chem.* **271**, 4266–72 (1996).

25. L. Lordier, A. Jalil, F. Aurade, F. Larbret, J. Larghero, N. Debili, W. Vainchenker, Y. Chang, Megakaryocyte endomitosis is a failure of late cytokinesis related to defects in the contractile ring and Rho/Rock signaling. *Blood* **112**, 3164–74 (2008).

26. M. Trakala, S. Rodríguez-Acebes, M. Maroto, C. E. Symonds, D. Santamaría, S. Ortega, M. Barbacid, J. Méndez, M. Malumbres, Functional reprogramming of polyploidization in megakaryocytes. *Dev. Cell* **32**, 155–67 (2015).

27. A. D. Sanchez, J. L. Feldman, Microtubule-organizing centers: From the centrosome to non-centrosomal sites. *Curr. Opin. Cell Biol.* **44**, 93–101 (2017).

28. L. Urbani, T. Stearns, The centrosome. *Curr. Biol.* **9**, R315–R317 (1999).

29. A. Kramer, B. Maier, J. Bartek, Centrosome clustering and chromosomal (in)stability: A matter of life and death. *Mol. Oncol.* **5**, 324–335 (2011).

30. B. Leber, B. Maier, F. Fuchs, J. Chi, P. Riffel, S. Anderhub, L. Wagner, A. D. Ho, J. L. Salisbury, M. Boutros, A. Krämer, Proteins required for centrosome clustering in cancer cells. *Sci. Transl. Med.* **2**, 33ra38 (2010).

31. M. Kwon, S. A. Godinho, N. S. Chandhok, N. J. Ganem, A. Azioune, M. Thery, D. Pellman, Mechanisms to suppress multipolar divisions in cancer cells with extra centrosomes. *Genes Dev.* **22**, 2189–2203 (2008).

32. D. L. Mercadante, W. A. Aaron, S. D. Olson, A. L. Manning, Cortical dynein drives centrosome clustering in cells with centrosome amplification. *Mol. Biol. Cell* **34**, ar63 (2023).

33. A. K. Weier, M. Homrich, S. Ebbinghaus, P. Juda, E. Miková, R. Hauschild, L. Zhang, T. Quast, E. Mass, A. Schlitzer, W. Kolanus, S. Burgdorf, O. J. Gruß, M. Hons, S. Wieser, E. Kiermaier, Multiple centrosomes enhance migration and immune cell effector functions of mature dendritic cells. *J. Cell Biol.* **221**, e202107134 (2022).

34. R. Philip, C. Fiorino, R. E. Harrison, Terminally differentiated osteoclasts organize centrosomes into large clusters for microtubule nucleation and bone resorption. *Mol. Biol. Cell* **33**, ar68 (2022).

35. J. Wu, K. Mikule, W. Wang, N. Su, P. Petteruti, F. Gharahdaghi, E. Code, X. Zhu, K. Jacques, Z. Lai, B. Yang, M. L. Lamb, C. Chuaqui, N. Keen, H. Chen, Discovery and mechanistic study of a small molecule inhibitor for motor protein KIFC1. *ACS Chem. Biol.* **8**, 2201–2208 (2013).

36. W. Zhang, L. Zhai, Y. Wang, R. J. Boohaker, W. Lu, V. V. Gupta, I. Padmalayam, R. J. Bostwick, E. L. White, L. J. Ross, J. Maddry, S. Ananthan, C. E. Augelli-Szafran, M. J. Suto, B. Xu, R. Li, Y. Li, Discovery of a novel inhibitor of kinesin-like protein KIFC1. *Biochem. J.* **473**, 1027–1035 (2016).

37. C. A. Watts, F. M. Richards, A. Bender, P. J. Bond, O. Korb, O. Kern, M. Riddick, P. Owen, R. M. Myers, J. Raff, F. Gergely, D. I. Jodrell, S. V. Ley, Design, synthesis, and biological evaluation of an allosteric inhibitor of HSET that targets cancer cells with supernumerary centrosomes. *Chem. Biol.* **20**, 1399–1410 (2013).

38. Z. Y. She, W. X. Yang, Molecular mechanisms of kinesin-14 motors in spindle assembly and chromosome segregation. *J. Cell Sci.* **130**, 2097–2110 (2017).

39. Y. Sekino, Q. T. Pham, K. Kobatake, H. Kitano, K. Ikeda, K. Goto, T. Hayashi, H. Nakahara, K. Sentani, N. Oue, W. Yasui, J. Teishima, N. Hinata, KIFC1 is associated with basal type, cisplatin resistance, PD-L1 expression and poor prognosis in bladder cancer. *J. Clin. Med.* **10**, 4837 (2021).

40. G. Fan, L. Sun, L. Meng, C. Hu, X. Wang, Z. Shi, C. Hu, Y. Han, Q. Yang, L. Cao, X. Zhang, Y. Zhang, X. Song, S. Xia, B. He, S. Zhang, C. Wang, The ATM and ATR kinases regulate centrosome clustering and tumor recurrence by targeting KIFC1 phosphorylation. *Nat. Commun.* **12**, 20 (2021).

41. Y. L. Wei, W. X. Yang, Kinesin-14 motor protein KIFC1 participates in DNA synthesis and chromatin maintenance. *Cell Death Dis.* **10**, 402 (2019).

42. Y. C. Lu, C. Sanada, J. Xavier-Ferrucio, L. Wang, P. X. Zhang, H. L. Grimes, M. Venkatasubramanian, K. Chetal, B. Aronow, N. Salomonis, D. S. Krause, The molecular signature of megakaryocyte-erythroid progenitors reveals a role for the cell cycle in fate specification. *Cell Rep.* **25**, 2083–2093.e4 (2018).

43. J. N. Thon, J. E. Italiano, Visualization and manipulation of the platelet and megakaryocyte cytoskeleton. *Methods Mol. Biol.* **788**, 109–125 (2012).

44. S. J. Doxsey, P. Stein, L. Evans, P. D. Calarco, M. Kirschner, Pericentrin, a highly conserved centrosome protein involved in microtubule organization. *Cell* **76**, 639–650 (1994).

45. J. L. Salisbury, K. M. Suino, R. Busby, M. Springett, Centrin-2 is required for centriole duplication in mammalian cells. *Curr. Biol.* **12**, 1287–1292 (2002).

46. G. D. Grant, K. M. Kedziora, J. C. Limas, J. G. Cook, J. E. Purvis, Accurate delineation of cell cycle phase transitions in living cells with PIP-FUCCI. *Cell Cycle* **17**, 2496–2516 (2018).

47. D. S. Shi, M. C. P. Smith, R. A. Campbell, P. W. Zimmerman, Z. B. Franks, B. F. Kraemer, K. R. Machlus, J. Ling, P. Kamba, H. Schwertz, J. W. Rowley, R. R. Miles, Z. J. Liu, M. Sola-Visner, J. E. Italiano, Jr., H. Christensen, W. H. A. Kahr, D. Y. Li, A. S. Weyrich, Proteasome function is required for platelet production. *J. Clin. Invest.* **124**, 3757–3766 (2014).

48. K. R. Machlus, S. K. Wu, D. J. Stumpo, T. S. Soussou, D. S. Paul, R. A. Campbell, H. Kalwa, T. Michel, W. Bergmeier, A. S. Weyrich, P. J. Blackshear, J. H. Hartwig, J. E. Italiano Jr, Synthesis and dephosphorylation of MARCKS in the late stages of megakaryocyte maturation drive proplatelet formation. *Blood* **127**, 1468–1480 (2016).

49. Y. Gao, E. Smith, E. Ker, P. Campbell, E. C. Cheng, S. Zou, S. Lin, L. Wang, S. Halene, D. S. Krause, Role of RhoA-specific guanine exchange factors in regulation of endomitosis in megakaryocytes. *Dev. Cell* **22**, 573–84 (2012).

50. V. Pannu, P. C. G. Rida, B. Celik, R. C. Turaga, A. Ogden, G. Cantuaria, J. Gopalakrishnan, R. Aneja, Centrosome-declustering drugs mediate a two-pronged attack on interphase and mitosis in supercentrosomal cancer cells. *Cell Death Dis.* **5**, e1538 (2014).

51. B. Rebacz, T. O. Larsen, M. H. Clausen, M. H. Rønnest, H. Löffler, A. D. Ho, A. Krämer, Identification of griseofulvin as an inhibitor of centrosomal clustering in a phenotype-based screen. *Cancer Res.* **67**, 6342–6350 (2007).

52. C. Sutterlin, A. Colanzi, The Golgi and the centrosome: Building a functional partnership. *J. Cell Biol.* **188**, 621–628 (2010).

53. J. N. Thon, M. T. Devine, A. Jurak Begonja, J. Tibbitts, J. E. Italiano, Jr., High-content live-cell imaging assay used to establish mechanism of trastuzumab emtansine (T-DM1)-mediated inhibition of platelet production. *Blood* **120**, 1975–1984 (2012).

54. J. S. Shin, S. W. Hong, S. L. Lee, T. H. Kim, I. C. Park, S. K. An, W. K. Lee, J. S. Lim, K. I. Kim, Y. Yang, S. S. Lee, D. H. Jin, M. S. Lee, Serum starvation induces G1 arrest through suppression of Skp2-CDK2 and CDK4 in SK-OV-3 cells. *Int. J. Oncol.* **32**, 435–439 (2008).

55. M. B. Mathews, R. M. Bernstein, B. R. Franza, Jr., J. I. Garrels, Identity of the proliferating cell nuclear antigen and cyclin. *Nature* **309**, 374–376 (1984).

56. C. Strassel, A. Eckly, C. Léon, S. Moog, J. P. Cazenave, C. Gachet, F. Lanza, Hirudin and heparin enable efficient megakaryocyte differentiation of mouse bone marrow progenitors. *Exp. Cell Res.* **318**, 25–32 (2012).

57. R. S. M. Wong, M. N. Saleh, A. Khelif, A. Salama, M. S. O. Portella, P. Burgess, J. B. Bussel, Safety and efficacy of long-term treatment of chronic/persistent ITP with eltrombopag: Final results of the EXTEND study. *Blood* **130**, 2527–2536 (2017).

58. N. Collins, S. J. Han, M. Enamorado, V. M. Link, B. Huang, E. A. Moseman, R. J. Kishton, J. P. Shannon, D. Dixit, S. R. Schwab, H. D. Hickman, N. P. Restifo, D. B. McGavern, P. L. Schwartzberg, Y. Belkaid, The bone marrow protects and optimizes immunological memory during dietary restriction. *Cell* **178**, 1088–1101.e15 (2019).

59. H. E. Allan, M. A. Hayman, S. Marcone, M. V. Chan, M. L. Edin, T. Maffucci, A. Joshi, L. Menke, M. Crescente, M. Mayr, D. C. Zeldin, P. C. Armstrong, T. D. Warner, Proteome and functional decline as platelets age in the circulation. *J. Thromb. Haemost.* **19**, 3095–3112 (2021).

60. A. Veninga, S. Handtke, K. Aurich, B. M. E. Tullemans, S. L. N. Brouns, S. L. Schwarz, F. C. J. I. Heubel-Moenen, A. Greinacher, J. W. M. Heemskerk, P. E. J. van der Meijden, T. Thiele, GPVI expression is linked to platelet size, age, and reactivity. *Blood Adv.* **6**, 4162–4173 (2022).

61. M. Grodzielski, J. A. Cidlowski, Glucocorticoids regulate thrombopoiesis by remodeling the megakaryocyte transcriptome. *J. Thromb. Haemost.* **21**, 3207–3223 (2023).

62. M. L. Steinhauser, B. A. Olenchock, J. O'Keefe, M. Lun, K. A. Pierce, H. Lee, L. Pantano, A. Klibanski, G. I. Shulman, C. B. Clish, P. K. Fazeli, The circulating metabolome of human starvation. *JCI Insight* **3**, e121434 (2018).

63. Y. Li, W. Lu, D. Chen, R. J. Boohaker, L. Zhai, I. Padmalayam, K. Wennerberg, B. Xu, W. Zhang, KIFC1 is a novel potential therapeutic target for breast cancer. *Cancer Biol. Ther.* **16,** 1316–1322 (2015).

64. S. Chatterjee, A. Sarkar, J. Zhu, A. Khodjakov, A. Mogilner, R. Paul, Mechanics of multicentrosomal clustering in bipolar mitotic spindles. *Biophys. J.* **119**, 434–447 (2020).

65. C. E. Miles, J. Zhu, A. Mogilner, Mechanical torque promotes bipolarity of the mitotic spindle through multi-centrosomal clustering. *Bull. Math. Biol.* **84**, 29 (2022).

66. Y. Ito, S. Nakamura, N. Sugimoto, T. Shigemori, Y. Kato, M. Ohno, S. Sakuma, K. Ito, H. Kumon, H. Hirose, H. Okamoto, M. Nogawa, M. Iwasaki, S. Kihara, K. Fujio, T. Matsumoto, N. Higashi, K. Hashimoto, A. Sawaguchi, K. I. Harimoto, M. Nakagawa, T. Yamamoto, M. Handa, N. Watanabe, E. Nishi, F. Arai, S. Nishimura, K. Eto, Turbulence activates platelet biogenesis to enable clinical scale ex vivo production. *Cell* **174**, 636–648.e18 (2018).

67. J. N. Thon, L. Mazutis, S. Wu, J. L. Sylman, A. Ehrlicher, K. R. Machlus, Q. Feng, S. Lu, R. Lanza, K. B. Neeves, D. A. Weitz, J. E. Italiano, Jr., Platelet bioreactor-on-a-chip. *Blood* **124**, 1857–1867 (2014).

68. H. Liu, J. Liu, L. Wang, F. Zhu, In vitro generation of megakaryocytes and platelets. *Front. Cell Dev. Biol.* **9**, 713434 (2021).

69. X. Sim, M. Poncz, P. Gadue, D. L. French, Understanding platelet generation from megakaryocytes: Implications for in vitro-derived platelets. *Blood* **127**, 1227–1233 (2016).

70. H. D. Schwer, P. Lecine, S. Tiwari, J. E. Italiano Jr, J. H. Hartwig, R. A. Shivdasani, A lineage-restricted and divergent beta-tubulin isoform is essential for the biogenesis, structure and function of blood platelets. *Curr. Biol.* **11**, 579–586 (2001).

71. C. Strassel, M. M. Magiera, A. Dupuis, M. Batzenschlager, A. Hovasse, I. Pleines, P. Guéguen, A. Eckly, S. Moog, L. Mallo, Q. Kimmerlin, S. Chappaz, J. M. Strub, N. Kathiresan, H. de la Salle, A. van Dorsselaer, C. Ferec, J. Y. Py, C. Gachet, C. Schaeffer-Reiss, B. T. Kile, C. Janke, F. Lanza, An essential role for α4A-tubulin in platelet biogenesis. *Life Sci. Alliance*, **2**, e201900309 (2019).

72. S. Kunert, I. Meyer, S. Fleischhauer, M. Wannack, J. Fiedler, R. A. Shivdasani, H. Schulze, The microtubule modulator RanBP10 plays a critical role in regulation of platelet discoid shape and degranulation. *Blood* **114**, 5532–5540 (2009).

73. A. Kramer, K. Neben, A. D. Ho, Centrosome replication, genomic instability and cancer. *Leukemia* **16**, 767–775 (2002).

74. Y. X. Xiao, W. X. Yang, KIFC1: A promising chemotherapy target for cancer treatment? *Oncotarget* **7**, 48656–48670 (2016).

75. M. J. Ausserlechner, P. Obexer, G. Böck, S. Geley, R. Kofler, Cyclin D3 and c-MYC control glucocorticoid-induced cell cycle arrest but not apoptosis in lymphoblastic leukemia cells. *Cell Death Differ.* **11**, 165–174 (2004).

76. I. Rogatsky, J. M. Trowbridge, M. J. Garabedian, Glucocorticoid receptor-mediated cell cycle arrest is achieved through distinct cell-specific transcriptional regulatory mechanisms. *Mol. Cell. Biol.* **17**, 3181–3193 (1997).

77. R. L. Mort, M. J. Ford, A. Sakaue-Sawano, N. O. Lindstrom, A. Casadio, A. T. Douglas, M. A. Keighren, P. Hohenstein, A. Miyawaki, I. J. Jackson, Fucci2a: A bicistronic cell cycle reporter that allows Cre mediated tissue specific expression in mice. *Cell Cycle* **13**, 2681–2696 (2014).

78. S. J. Barsam, B. Psaila, M. Forestier, L. K. Page, P. A. Sloane, J. T. Geyer, G. O. Villarica, M. M. Ruisi, T. B. Gernsheimer, J. H. Beer, J. B. Bussel, Platelet production and platelet destruction: Assessing mechanisms of treatment effect in immune thrombocytopenia. *Blood* **117**, 5723–5732 (2011).

79. S. Brandhorst, I. Y. Choi, M. Wei, C. W. Cheng, S. Sedrakyan, G. Navarrete, L. Dubeau, L. P. Yap, R. Park, M. Vinciguerra, S. di Biase, H. Mirzaei, M. G. Mirisola, P. Childress, L. Ji, S. Groshen, F. Penna, P. Odetti, L. Perin, P. S. Conti, Y. Ikeno, B. K. Kennedy, P. Cohen, T. E. Morgan, T. B. Dorff, V. D. Longo, A periodic diet that mimics fasting promotes multi-system regeneration, enhanced cognitive performance, and healthspan. *Cell Metab.* **22**, 86–99 (2015).

80. V. D. Longo, M. P. Mattson, Fasting: Molecular mechanisms and clinical applications. *Cell Metab.* **19**, 181–192 (2014).

81. C. W. Cheng, G. B. Adams, L. Perin, M. Wei, X. Zhou, B. S. Lam, S. da Sacco, M. Mirisola, D. I. Quinn, T. B. Dorff, J. J. Kopchick, V. D. Longo, Prolonged fasting reduces IGF-1/PKA to promote hematopoietic-stem-cell-based regeneration and reverse immunosuppression. *Cell Stem Cell* **14**, 810–823 (2014).

82. E. Mikaelsdottir, G. Thorleifsson, L. Stefansdottir, G. Halldorsson, J. K. Sigurdsson, S. H. Lund, V. Tragante, P. Melsted, S. Rognvaldsson, K. Norland, A. Helgadottir, M. K. Magnusson, G. B. Ragnarsson, S. Y. Kristinsson, S. Reykdal, B. Vidarsson, I. J. Gudmundsdottir, I. Olafsson, P. T. Onundarson, O. Sigurdardottir, E. L. Sigurdsson, G. Grondal, A. J. Geirsson, G. Geirsson, J. Gudmundsson, H. Holm, S. Saevarsdottir, I. Jonsdottir, G. Thorgeirsson, D. F. Gudbjartsson, U. Thorsteinsdottir, T. Rafnar, K. Stefansson, Genetic variants associated with platelet count are predictive of human disease and physiological markers. *Commun. Biol.* **4**, 1132 (2021).

83. P. Vijey, B. Posorske, K. R. Machlus, In vitro culture of murine megakaryocytes from fetal liver-derived hematopoietic stem cells. *Platelets* **29**, 583–588 (2018).

84. T. Heib, C. Gross, M.-L. Müller, D. Stegner, I. Pleines, Isolation of murine bone marrow by centrifugation or flushing for the analysis of hematopoietic cells - A comparative study. *Platelets* **32**, 601–607 (2021).

85. T. Stepanova, J. Slemmer, C. C. Hoogenraad, G. Lansbergen, B. Dortland, C. I. de Zeeuw, F. Grosveld, G. van Cappellen, A. Akhmanova, N. Galjart, Visualization of microtubule growth in cultured neurons via the use of EB3-GFP (end-binding protein 3-green fluorescent protein). *J. Neurosci.* **23**, 2655–2664 (2003).

86. H. Umeshima, T. Hirano, M. Kengaku, Microtubule-based nuclear movement occurs independently of centrosome positioning in migrating neurons. *Proc. Natl. Acad. Sci. U.S.A.* **104,** 16182–16187 (2007).

87. S. L. French, P. Vijey, K. W. Karhohs, A. R. Wilkie, L. J. Horin, A. Ray, B. Posorske, A. E. Carpenter, K. R. Machlus, J. E. Italiano Jr, High-content, label-free analysis of proplatelet production from megakaryocytes. *J. Thromb. Haemost.* **18**, 2701–2711 (2020).

88. T. Kawamoto, M. Shimizu, A method for preparing 2- to 50-micron-thick fresh-frozen sections of large samples and undecalcified hard tissues. *Histochem. Cell Biol.* **113**, 331–339 (2000).

89. P. Wadsworth, TPX2. *Curr. Biol.* **25**, R1156–R1158 (2015).

90. E. Willems, M. Dedobbeleer, M. Digregorio, A. Lombard, P. N. Lumapat, B. Rogister, The functional diversity of Aurora kinases: A comprehensive review. *Cell Div.* **13**, 7 (2018).

91. G. de Carcer, G. Manning, M. Malumbres, From Plk1 to Plk5: Functional evolution of Polo-like kinases. *Cell Cycle* **10**, 2255–2262 (2011).

92. M. Venere, C. Horbinski, J. F. Crish, X. Jin, A. Vasanji, J. Major, A. C. Burrows, C. Chang, J. Prokop, Q. Wu, P. A. Sims, P. Canoll, M. K. Summers, S. S. Rosenfeld, J. N. Rich, The mitotic kinesin KIF11 is a driver of invasion, proliferation, and self-renewal in glioblastoma. *Sci. Transl. Med.* **7**, 304ra143 (2015).

93. A. L. Manning, N. J. Ganem, S. F. Bakhoum, M. Wagenbach, L. Wordeman, D. A. Compton, The kinesin-13 proteins Kif2a, Kif2b, and Kif2c/MCAK have distinct roles during mitosis in human cells. *Mol. Biol. Cell* **18**, 2970–2979 (2007).

94. S. Srivastava, D. Panda, A centrosomal protein STARD9 promotes microtubule stability and regulates spindle microtubule dynamics. *Cell Cycle* **17**, 2052–2068 (2018).

95. T. Chinen, K. Yamazaki, K. Hashimoto, K. Fujii, K. Watanabe, Y. Takeda, S. Yamamoto, Y. Nozaki, Y. Tsuchiya, D. Takao, D. Kitagawa, Centriole and PCM cooperatively recruit CEP192 to spindle poles to promote bipolar spindle assembly. *J. Cell Biol.* **220**, e202006085 (2021).
